# Influence of environmental and anthropogenic factors on forest patch composition and structure in North Wollo Zone, Amhara region, Ethiopia

**DOI:** 10.1371/journal.pone.0332831

**Published:** 2025-09-23

**Authors:** Mulugeta Alemu, Bikila Warkineh, Ermias Lulekal, Zemede Asfaw

**Affiliations:** 1 Addis Ababa University, College of Natural and Computational Sciences, Department of Plant Biology and Biodiversity Management, Addis Ababa, Ethiopia; 2 Lemi Kura Manufacturing College, Department of Urban Agriculture, Addis Ababa, Ethiopia; Qingdao Agricultural University, CHINA

## Abstract

Ethiopia’s vegetation is increasingly threatened by anthropogenic activities and natural factors, leading to forest degradation and fragmentation. This study analyzed the composition and structure of woody species in the Gerado, Micha, and Mekelet forest patches in the North Wollo Zone, Ethiopia. Data were collected from 95 systematically laid plots (20 x 20 m²) along elevation gradients, sampling trees and shrubs with a diameter at breast height (DBH) ≥ 2.5 cm and height ≥ 2 m. Species diversity and richness were assessed using the Shannon-Wiener diversity index, while plant communities and their relationships with environmental variables were analyzed using hierarchical clustering and Redundancy Analysis (RDA) in R software. A total of 55 woody species, from 46 genera and 31 families, were recorded. Fabaceae was the most species-rich family (12 species). Four distinct plant community types were identified (*R = 0.4703, p ≤ 0.001*), with altitude, slope, and tree cutting significantly influencing community composition (*p ≤ 0.05*). The dominant species across all patches were *Dodonaea viscosa* subsp. *angustifolia*, *Olea europaea* L. subsp. *cuspidata*, and *Vachellia sieberiana*. Anthropogenic disturbances, such as tree cutting, firewood collection, charcoal production, and grazing, were key factors affecting vegetation structure. The findings highlight the need for community-based conservation strategies tailored to the unique ecological and socio-economic conditions of each forest patch to improve ecosystem resilience and sustainability.

## Introduction

Forest ecosystems play multiple roles at local, national, and global levels through the provision of various goods and services including food, medicine, ecological and sociocultural advantages [1]. Tropical forests are known for their high levels of biodiversity, which support numerous living species and constitute the complex webs of life [2].

Ethiopia is characterized by a wide range of ecological, edaphic, and climatic conditions that account for the wide diversity of biological resources, both in terms of flora, faunal and microbial wealth [3,4]. Its complex topography and environmental heterogeneity foster a wide array of life forms [5,6]. The country is in tropics with diverse vegetation types, including forests and woodland vegetation [7]. Even though the diverse ecosystems of Ethiopia have endowed the country with various biological wealth of plants and animals, for a variety of reasons, these resources are under severe threat [8]. Population growth, combined with human activities such as resource extraction, burning, and land conversion, significantly impacts natural forests, alongside natural factors [9]. Ethiopia’s biodiversity faces additional threats from habitat loss, invasive species, unsustainable resource use, climate change, and pollution, compounded by poor stakeholder coordination [7,10]. These pressures, along with agricultural expansion, overgrazing, and unsustainable timber and fuel wood extraction, are primary drivers of deforestation [11–15], leaving only small forest remnants in the northeast and northwest regions, which continue to face increasing anthropogenic threats.

**Fig 1:**
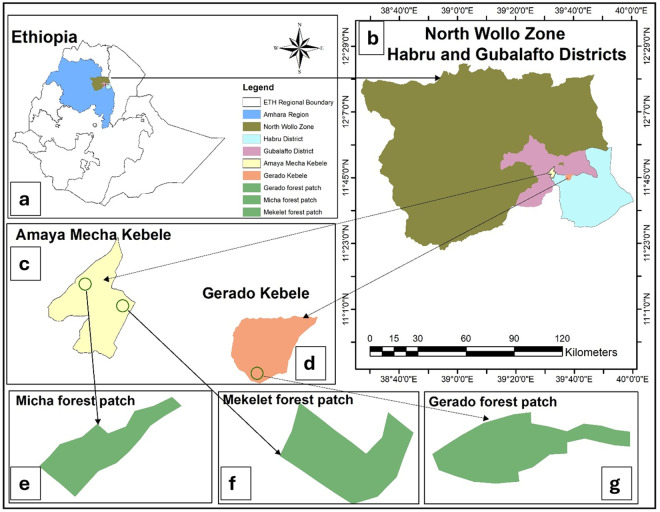
Map of Ethiopia showing the location of the three forest patches in the Habru District and its environs, North Wollo Zone, Amhara Regional State. The map was prepared by the authors using ArcGIS 10.5. Administrative boundary shapefiles were obtained from the Africa GeoPortal (https://ethiopia.africageoportal.com/). No copyrighted imagery was used. (a) Ethiopia with regional boundaries and study area. (b) North Wollo Zone, highlighting Habru and Gubalafto districts, where the study forest patches are located. (c) Amaya Mecha Kebele (Village), where the Micha and Mekelet forest patches are situated. (d) Gerado Kebele within Habru District, the location of the Gerado forest patch. (e) Micha forest patch in Amaya Mecha Kebele. (f) Mekelet forest patch in Amaya Mecha Kebele. (g) Gerado forest patch in Gerado Kebele.

The vegetation type of the study area is classified as dry Afromontane forests (DAF) of Ethiopia [16]. Within the Wollo Floristic region, the Gerado, Micha, and Mekelet forests patches (Fig 1), are experiencing the aforementioned anthropogenic pressures. Thus, understanding not only the ecological information on floristic composition and distribution of plant species but also evaluating the human impact on community structure and vegetation dynamics is essential. Such integrated knowledge is critical for conservation planning, ecosystem restoration, and the development of sustainable management practices. Given this, the present study aims to examine the status of the floristic composition, vegetation structure and plant community types along with environmental and anthropogenic disturbance factors of Gerado, Micha and Mekelet forest patches in the North Wollo Zone of Amhara region, Ethiopia.

## Materials and methods

### Description of the study area

This study was carried out in the Gerado, Mekelet, and Micha forest patches located in the Habru and Gubalafto districts, North Wollo Zone, Amhara Regional State, Ethiopia (Fig 1). The forest patches fall within the Dry Evergreen Afromontane Forest and Grassland Complex, typically dominated by *Afrocarpus falcatus* (Thunb.) C.N.Page (Podocarpaceae) and *Juniperus procera* Hochst. ex Endl. (Cupressaceae), with *Olea europaea* L. subsp. *cuspidata* (Wall. & G.Don) Cif. (Oleaceae) [17,18]. The study area spans an altitudinal range of 1430–2800 masl. The agroecological zones include warm semi-arid (Kolla), cool and humid (Dega), and cool sub-humid (Woina Dega) areas, with an average annual temperature of 20.1 °C and a bimodal rainfall distribution of approximately 1045 mm/year [19–22].

### Sampling design and vegetation data collection

For the study, a systematic sampling approach with restrictions based on Ellenberg and Mueller-Dombois [23], complemented by the Braun-Blanquet scale as modified by Van der Maarel [24] was used to estimate the cover-abundance value of all woody plants. A total of 95 quadrats (400 m² each) were sampled across all forest patches along elevation gradients. In total, 26 transect lines were established, and topographic variables such as altitude, latitude, and longitude were recorded for each quadrat using a Geographic Positioning System (GPS). The number of sampling plots, as well as the distances between plots and transect lines, varied among the forest patches based on factors such as forest cover, altitude, and habitat variability, following the methods recommended by Kent and Coker [25]. In areas with dense forest cover, plots were spaced at shorter intervals (100 m) to account for high species turnover, while in more open or homogeneous areas, intervals were increased up to 200 m. At higher altitudes, closer spacing was used to capture micro-environmental variability, whereas at lower altitudes and in more uniform habitats, wider spacing was applied. These adjustments followed ecological sampling protocols to ensure representative coverage of environmental gradients.

The percent cover of each trees and shrubs in a 400 m² plot was visually estimated by the same person to maintain consistency across all plots (S1 File). The scientific and local names, abundance and habits of each woody species were recorded in each plot. Additionally, the diameter at breast height (≥2.5 cm) and ≥2 m height thresholds was measured for all woody species using a tree caliper, and plant heights were measured with a calibrated stick. Environmental variables such as altitude, longitude, latitude, and aspect of each quadrat, were measured using GPS. For analytical clarity, aspect measurements were codified in accordance with the established system [26]. This coding translated the cardinal and intercardinal directions into numerical values: North (N) as 0, Northeast (NE) as 1, East (E) as 2, Southeast (SE) as 3, South (S) as 4, Southwest (SW) as 3.25, West (W) as 2.5, and Northwest (NW) as 1.25 (S2 File). This coding was used to quantify the aspect (direction of slope) for each plot, allowing us to incorporate it as a variable in the statistical analysis. The impact of anthropogenic disturbance (cutting, charcoal production, agricultural expansion, firewood collection, grazing and browsing) was recorded in the quadrats using a 0–3 subjective ordinal scale as 3 = heavy, 2 = moderate, 1 = low and 0 = nil [27–29]. Voucher specimens of plants were collected, and the preliminary identification was executed using manuals in the field and the collected specimens were pressed, dried, and brought to the National Herbarium (ETH) of Addis Ababa University, and determinations were undertaken. The determination was performed using the taxonomic keys of the Flora of Ethiopia and Eritrea (Volume 1–8), followed by comparisons with authenticated specimens in the ETH, and finally confirmed by a taxonomic expert at Addis Ababa University. Following identification, each specimen was assigned a voucher number and detailed information was recorded, including family, species, vernacular names, collection date, and collection site. These vouchered specimens were then deposited at the ETH for future reference.

### Data analysis

In this study, we applied a combination of univariate and multivariate statistical techniques to evaluate species diversity, community composition, vegetation structure, and the influence of environmental and anthropogenic factors. Diversity indices were used to quantify richness and evenness across the study sites, while community classification and ordination techniques helped identify distinct vegetation assemblages and their ecological drivers. Structural parameters such as basal area, density, frequency, and Importance Value Index (IVI) were calculated to assess dominance patterns and regeneration status. Furthermore, disturbance indicators were incorporated into the analyses to explore the extent to which human activities shape forest composition and structure. This integrated analytical approach provided a comprehensive understanding of the ecological dynamics of the three forest patches.

**Plant diversity analysis:** The floristic diversity of the study area was measured using the Shannon-Wiener diversity index to quantify species richness and evenness [25]. The Shannon-Wiener diversity index was calculated as follows:


$${𝐇}^{\prime }=-\sum_{i=1}^{s}pi𝐈𝐧pi$$

Where, **H’ **= Shannon-Wiener Diversity Index; **S** = The number of species; **Pi** = the proportion of individuals or the abundance of the i^th^ species expressed as a proportion of total cover; **In** = logbase_n_

The Shannon’s evenness or equitability of the woody species was measured as:


$$J=\;\frac{H^{\prime }}{H^{\prime }max}$$

Where: **J **= Evenness, **H’ = **Shannon-Wiener Diversity Index; **H’ max = **Ins, where s is number of species

**Rarefaction analysis:** To account for potential bias due to unequal sampling effort among forest patches, we conducted species rarefaction analyses using both the vegan and iNEXT packages in R [30,31]. First, we applied sample-based rarefaction curves (rarefy and rarecurve functions in vegan) to evaluate cumulative species richness with increasing numbers of quadrats. Second, we used the iNEXT framework to generate individual-based rarefaction and extrapolation curves with 95% confidence intervals, enabling direct comparison of observed and estimated species richness under different disturbance regimes. This dual approach allowed us to assess whether observed richness patterns were artifacts of sampling effort or represented true ecological contrasts. The full R scripts and outputs are provided as supplementary information (S9 File).

**Plant community classification:** Plant community types of forest patches were classified using Agglomerative hierarchical clustering analysis using R programming language version 4.2.2 [32] using vegan package [33]. The input matrix for plant community classification was structured using cover-abundance values, which served as the basis for the clustering analysis [34]. Considering all forest patches as one, four plant community types were identified using Ward’s method of hierarchical clustering technique as recommended by Kent and Coker [25]. In this study, the optimal number of clusters was determined using the elbow method (S8 File). This method involves plotting the within-group sum of squares (WSS) against the number of clusters and identifying the point where the rate of decrease sharply changes, known as the elbow point. For our dataset, the elbow point was observed at four clusters, indicating this as the optimal number for our k-means clustering analysis [35]. Synoptic values were computed as the result of average cover-abundance values of species and their frequency in a specific community type. Thus, two characteristic species with high synoptic cover-abundance values were used to name the plant community types [25]. Multi-Response Permutation Procedure (MRPP) test was also used to test whether there is significant difference between the clusters [36]. Floristic similarity analysis between forest patches of the study area were computed using Sorensen’s similarity coefficient as follows:


$$Ss=\;\frac{2𝐚}{2𝐚+𝐛+𝐜}$$

Where: Ss = Sorensen’s similarity coefficient; a = number of species common to both forest patches compared; b = number of species in one forest patch; c = number of species in the other forest

Detrended Correspondence Analysis (DCA) was utilized to discern the appropriateness of Canonical Correspondence Analysis (CCA) or Redundancy Analysis (RDA) for examining the interplay between plant communities and environmental variables [37]. Given the first DCA axis lengths (DCA1: 2.7942), which indicates that the data likely exhibits linear rather than unimodal species responses to the gradients (Table 1). Therefore, the outcomes of DCA indicated that RDA was the most suitable method, subsequently guiding the exploration of associations between all documented plant species within the communities and various environmental factors, including altitude, slope, aspect, and anthropogenic disturbances. Additionally, the Adonis 2 test (Table 2) was utilized to further investigate the impact of these environmental factors on the distribution of plant communities following the method described by Anderson et al. [38].

**Table 1:** Detrended correspondence analysis (DCA) result of vegetation data.

	DCA1	DCA2	DCA3	DCA4
Eigenvalues	0.2528	0.2150	0.1750	0.1815
Decorana values	0.3167	0.2421	0.1914	0.1533
Axis lengths	2.7942	2.8080	2.3104	2.0698

**Table 2:** Outcomes of the Adonis 2 test on significant environmental variables.

	Df	SumsOfSqs	MeanSqs	F.Model	R2	Pr(>F)
Slope	1	1.3412	1.34116	9.2263	0.08536	0.001 ***
Aspect	1	0.2709	0.27090	1.8636	0.01724	0.053.
Cutting	1	0.4815	0.48151	3.3125	0.03065	0.001 ***
Grazing	1	0.1468	0.14679	1.0098	0.00934	0.420
Browsing	1	0.1483	0.14830	1.0202	0.00944	0.413
Altitude	1	0.5818	0.58175	4.0021	0.03703	0.001 ***
FWC	1	0.2552	0.25519	1.7556	0.01624	0.068.
CP	1	0.0457	0.04571	0.3145	0.00291	0.969
AE	1	0.0844	0.08438	0.5805	0.00537	0.799
Residuals	85	12.3559	0.14536		0.78642	
Total	95	15.7116			1.00000	

FWC = Firewood collection; CP = Charcoal production; AE = Agricultural production;

Signif. Codes: 0 ‘***’ 0.001 ‘**’ 0.01 ‘*’ 0.05 ‘.’ 0.1 ‘’ 1

**Anthropogenic disturbance:** Human disturbances were evaluated through systematic field-based visual assessments across each 20 m × 20 m plot. We documented five key disturbance indicators: (1) tree cutting (fresh stumps, logged trunks), (2) firewood collection (branch removal, stacked fuelwood), (3) charcoal production (kiln sites, charred remains), (4) livestock grazing (dung, trampling, browsing damage), and (5) agricultural expansion (crop encroachment at forest edges). [27].

The impact of disturbance was quantified using the variable scores recorded in each sample plot for all forest patches. The degree of disturbance was estimated based on the average score value of anthropogenic factors, following methodologies adapted from prior Ethiopian forest studies [27–29,39]. Then, the disturbance scores for each forest patch were assigned to reflect the highest rate of disturbance and the absence of disturbance. Limitations of this approach include its reliance on qualitative observation, which may introduce subjectivity despite efforts to standardize scoring across all plots. Future studies would benefit from integrating remote sensing data or community-based participatory assessments to improve accuracy.

**Vegetation structure analysis:** The structural analysis of vegetation was described using species density, DBH, height, basal area (BA) per hectare, frequency, and important value index (IVI), following Tamrat Bekele [40]. The frequency distribution of a species was computed as the proportion of samples within which a species is found. The dominance of key woody species was measured using the Importance Value Index (IVI), considering three factors: relative density, relative dominance, and relative frequency [23,25].

Different formulas that are important in conducting structural analysis are described below:


$$Density\;(D)=\frac{The\;number\;of\;individuals\;of\;species}{Unit\;area\;of\;the\;quadrat\;(hectare)}$$

$$Relative\;density\;(RD)=\frac{Number\;of\;individuals\;of\;species\;A}{Total\;number\;of\;individuals\;of\;all\;species}\times \text{1}\text{0}\text{0}$$

$$Frequency\;(F)=\frac{Number\;of\;sampling\;units\;or\;quadrats\;in\;which\;species\;A\;recorded}{Total\;number\;of\;\;sampling\;units\;or\;quadrats\;sampled}$$

$$Relative\;frequency\;(RF)=\frac{frequency\;of\;single\;species\;}{Sum\;of\;frequencies\;of\;all\;species}\times \text{1}\text{0}\text{0}$$

$𝐁asal\;Area\;(BA)=\frac{\pi d^{\text{2}}\;}{\text{4}}$; Where, π = 3.14; d = DBH (m)


$$Dominance\;(Do)=\frac{Area\;covered\;by\;a\;species\;(BA)}{Sum\;of\;all\;quadrats\;area\;in\;hectare}$$

$$Relative\;dominance\;\left(RDo\right)=\frac{Basal\;area\;of\;a\;single\;species}{Total\;basal\;area\;of\;all\;species}\times \text{1}\text{0}\text{0}$$

Importance Value Index (IVI)=RD + RDo+ RF

Where, IVI = Importance Value Index; RD = Relative Density; RDo = Relative Dominance and RF = Relative Frequency

## Results

### Composition of woody species in the studied forest patches

**Fig 2:**
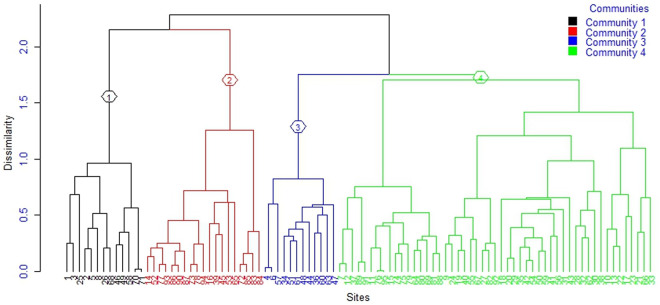
Dendrogram showing the four plant community types of the forest patches.

A total of 55 woody plant species belonging to 31 families and 46 genera were recorded in the study area (Table 3 and S3 File). The highest species richness was recorded for the Fabaceae family 12 (21.8%), followed by the Anacardiaceae, Euphorbiaceae, Malvaceae, and Moraceae with 3 (5.5%) each (Fig 2). Among the total woody species recorded in the study areas, 28 (51%) were trees, and 27 (49%) were found to be shrubs. Sample-based rarefaction (vegan) indicated that species richness differed markedly among the three forest patches. The highest richness among the forest patches was recorded in Gerado forest, followed by Micha and Mekelet forest patches. Gerado has 50 woody plant species belonging to 44 genera and 32 families. Micha has 32 woody plant species belonging to 27 genera and 19 families. Mekelet also has 21 plant species belonging to 20 genera and 16 families.

**Table 3:** Total number of species, genera, families, and growth forms per forest patch.

Patch name	Coordinates	Area (ha)	Altitudinal range (masl)	No. of plots	Number of transects	Richness	Family	Genera	Growth form	
									Tree	Shrub
Gerado	11^o^44’13.55“N 39^o^37’56.61”E	75	1925–2226	43	12	50	32	44	24	26
Micha	11^o^47’11.24“N & 39^o^31’58.9”E	44.2	1997–2226	28	9	32	19	27	14	18
Mekelet	11^o^46’31.02“N & 39^o^33’23.72”E	92	2059 to 2413	24	5	21	16	20	9	12

Individual-based rarefaction and extrapolation (iNEXT) confirmed these findings (S8 File). Under both girdled and logged disturbance regimes, the group corresponding to Gerado (code 0) consistently exhibited the highest richness, with extrapolated curves indicating further species discovery at larger sample sizes. In contrast, the Mekelet group (code 2) showed very limited richness, with curves flattening early, while Micha (code 1) displayed intermediate diversity. Together, the vegan and iNEXT analyses indicate that species richness patterns among the forest patches are robust, with Gerado retaining relatively high diversity, Micha showing moderate richness, and Mekelet reflecting significant biodiversity loss.

### Plant communities of the studied forest patches

In this study, four plant community types were identified (Fig 2). There is a statistically significant difference (*R = 0.4703, p ≤ 0.001*) in floristic compositions of the woody species among the four identified plant community types in all forest patches based on the Multi-response Permutation Procedures (MRPP) test (S7 File). Each plant community types (C1-C4) were named after the two identifying characteristics of species in each group (Table 4).

**Table 4:** Characteristic species in each community type of the studied forest patches based on the average synoptic cover-abundance value (S6 File).

Characteristics species	Community types			
	C1	C2	C3	C4
*Hesperocyparis lusitanica*	**1.92**	0.07	0.04	0.53
*Juniperus procera*	**7**	0.67	1.38	0.21
*Pittosporum viridiflorum*	1.62	**5.07**	2.9	0.53
*Dodonaea viscosa* subsp. *angustifolia*	2.92	**4.93**	4.21	3.47
*Olea europaea* L. subsp. *cuspidata*	2.54	2.13	**5.52**	3.16
*Allophylus abyssinicus*	0.77	0.27	**1.4**	0.21
*Vachellia sieberiana*	4.08	5.4	3.15	**5.63**
*Carissa spinarum*	0	1.2	1.0	**0.68**

Key: C1: community type 1; C2: community type 2; C3: community type 3; C4: community type 4.

The detailed descriptions of the community types identified from Gerado, Micha and Mekelet forest patches are given below.

(i) ***Hesperocyparis lusitanica – Juniperus procera* community type (C1):** This community type represents 13 plots and 25 species found between the altitudinal ranges of 1925–2204 m.a.s.l. The dominant tree and shrub species of this community type include *Juniperus procera, Vachellia sieberiana, Dodonaea viscosa* subsp. *angustifolia, Olea europaea* L. subsp. *cuspidata, Hesperocyparis lusitanica, Eucalyptus camaldulensis, Euclea racemosa, Pittosporum viridiflorum* and *Osyris lanceolata.*
(ii) ***Pittosporum viridiflorum – Dodonaea viscosa* subsp. *angustifolia* community Type (C2):** This community type represents 19 plots and 28 species between the altitudinal ranges of 1929–2178 m.a.s.l. The dominant tree and shrub species of this community type include *Vachellia sieberiana, Pittosporum viridiflorum***,** *Dodonaea viscosa* subsp. *angustifolia, Searsia retinorrhoea, Olea europaea* L. subsp. *cuspidata*, *Carissa spinarum, Vachellia etbaica* and *Euclea racemosa.*
(iii) ***Olea europaea* L. subsp. *cuspidata – Allophylus abyssinicus* community type (C3):** This community type represents 12 plots and 45 species between the altitudinal ranges of 1946–2226 m.a.s.l. The dominant tree and shrub species of this community type include *Olea europaea* L. subsp. *cuspidata, Dodonaea viscosa* subsp**. angustifolia, Vachellia sieberiana, Pittosporum viridiflorum, Allophylus abyssinicus,* *Juniperus procera, Carissa spinarum, Euphorbia abyssinica** and *Euclea racemosa.*
(iv) ***Vachellia sieberiana – Carissa spinarum* community type (C4):** This community type represents 51 plots and 31 species between the altitudinal ranges of 1937–2413 m.a.s.l. The dominant tree and shrub species of this community type include *Vachellia sieberiana, Dodonaea viscosa* subsp. *angustifolia, Olea europaea* L. subsp. *cuspidata, Carissa spinarum, Eucalyptus camaldulensis,* and *Osyris lanceolata.*

### Plant community relationship with environmental variables

**Fig 3:**
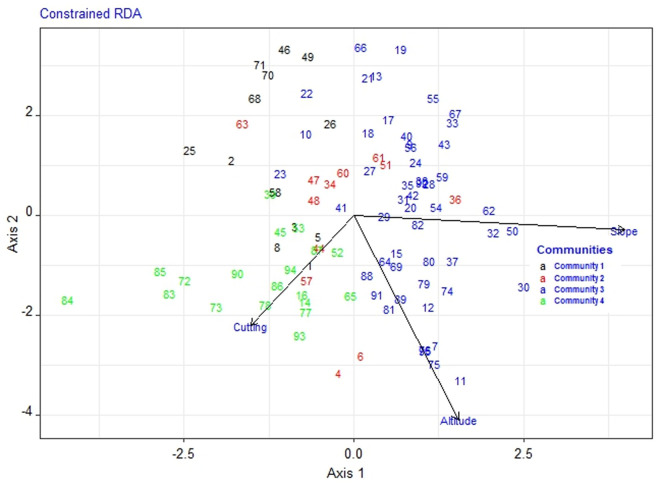
Redundancy Analysis (RDA) showing the relationship between plant community types and significant environmental variables (p≤ 0.05), including slope, altitude, and cutting pressure. Plots 1–28 were collected from Micha, Plots 29–71 from Gerado, and Plots 72–95 from Mekelet. The colors of the numbers indicate the community type classification. Full plot details are provided in the Supporting Information (S2 File).

The RDA results showed that slope, altitude and cutting had significant impacts (*p ≤ *0.05) on the composition and distribution of community types (Fig 3). The correspondence between forest patches and plant communities is now clearly illustrated. Community Type 1 (C1) is represented by both Micha (7 plots) and Gerado (6 plots). Community Type 2 (C2) is found in Gerado (4 plots) and Mekelet (15 plots). Community Type 3 (C3) consists of 2 plots from Micha and 10 plots from Gerado. Finally, Community Type 4 (C4) is the most widely distributed, covering Micha (17 plots), Gerado (22 plots), and Mekelet (12 plots). This distribution reflects ecological variability across the patches, with certain forest patches supporting multiple plant community types due to differences in environmental factors such as elevation and habitat diversity. Moreover, Communities 1 and 2 were primarily associated with lower elevations, suggesting that certain species thrive under less steep and more accessible conditions. Community 3 was linked to steeper slopes and showed the broadest altitudinal variation, indicating adaptation to topographically complex environments. Community 4 was distinctly associated with higher cutting pressure, reinforcing its identity as a disturbance-adapted group. These interactions underscore how environmental gradients and human disturbance jointly shape vegetation patterns across fragmented forest patches.

### Floristic diversity indices

The results reveal that Gerado has the highest species richness, followed by Micha and then Mekelet. Among the forest patches, the highest diversity was observed in community type 3 (2.752), followed by community type 2 (2.56). The Shannon evenness value was also in the same order with little variations, the highest being for community type 1 (0.779) and community type 2 (0.777) and the least evenness was for community type 4 (Table 5).

**Table 5:** Species richness and diversity indices of plant communities identified in the forest patches in the study area.

Forest Patch	Communities	Richness	H	Shannon Evenness	Correspondence between the forest patches and community
Gerado, Micha and Mekelet (All in one)	C1	25	2.506	0.779	Micha 7 plots; Gerado 6 plots
	C2	27	2.560	0.777	Gerado 4 plots; Mekelet 15 plots
	C3	45	2.752	0.723	Micha 2 plots; Gerado 10 plots
	C4	31	2.471	0.720	Micha 17 plots; Gerado 22 plots; Mekelet 12 plots

### Floristic composition similarities between forest patches

**Fig 4:**
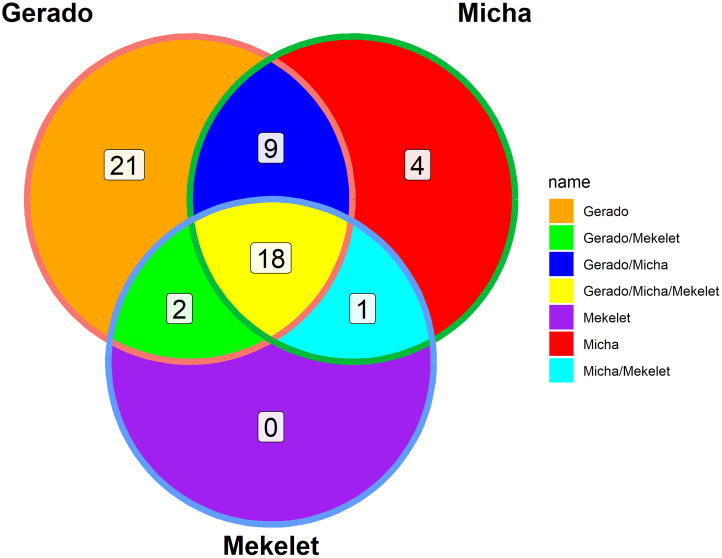
Venn-Diagram of species in Gerado, Micha and Mekelet forest patches.

The similarities between forest patches were generally strong. Accordingly, Mekelet and Micha patch forests exhibited the highest similarity with Ss = 0.71. Relatively the least similarity was observed between Gerado and Mekelet patch forests (Ss = 0.56). The analysis revealed that the Mekelet Forest Patch did not possess its own distinct species (Fig 4), a pattern that may be linked to the high levels of disturbance observed in the area. As shown in Table 6, disturbances such as overgrazing and firewood collection were more pronounced in Mekelet compared to other patches.

**Table 6:** Mean value of anthropogenic disturbances along the three forest patches.

Patch name and total number of plots	Reported plots and score value (mean)	Cutting	Charcoal production	Agricultural expansion	Firewood collection	Grazing	Browsing	Total
Gerado, 43 plots	No. of plots	25	0	13	14	6	9	
	Mean	1	0.04	0.82	0.46	0.39	0.25	2.96
Micha, 28 plots	No. of plots	19	1	13	8	11	7	
	Mean	0.67	0.00	0.56	0.35	0.19	0.26	2.02
Mekelet, 24 plots	No. of plots	22	5	9	11	3	4	
	Mean	1.71	0.38	0.67	0.75	0.17	0.29	3.96

Note: 0 indicates there was no disturbance was observed, rather than missing.

### Anthropogenic disturbances in the forest patches

The highest average score was for Mekelet forest patch (3.96), followed by Gerado and Micha. The intensity of tree cutting and firewood collection significantly contribute to the elevated disturbance level in Mekelet forest patch (Table 6). In Gerado and Micha cutting and agricultural expansion were the most important impacts.

### Vegetation structure

#### Density.

In the Gerado forest patch, the most abundant species is *Dodonaea viscosa* subsp. *angustifolia*, followed by *Olea europaea* L. subsp. *cuspidata*. The species with the highest RD is also *Dodonaea viscosa* subsp. *angustifolia*, indicating its significant presence and ecological influence within the Gerado patch. Micha’s species distribution is similar to Gerado’s, with *Dodonaea viscosa* subsp. *angustifolia* and *Olea europaea* L. subsp. *cuspidata* again being the most abundant. However, *Dodonaea viscosa* subsp. *angustifolia* has the highest RD here, which could suggest it has a more significant impact on the ecosystem’s structure in Micha. Mekelet shows a different pattern, where *Vachellia sieberiana* has the highest number of individuals and RD, indicating its dominance in this patch (S5 File).

#### Frequency.

The Gerado and Micha share a similar species hierarchy, with these species occurring in over 85% of plots, reflective of their adaptability and potential influence on local biodiversity. *Dodonaea viscosa* subsp. *angustifolia* and *Vachellia sieberiana* are the most frequent species, both occurring in 95.35% of plots, with the highest RF of 10.17, indicating their widespread presence and possible dominance in the forest structure in Gerado. Other species like *Pittosporum viridiflorum*, *Olea europaea* L. subsp. *cuspidata*, and *Euclea racemosa* L. also show high frequency, suggesting a diverse but specific set of dominant species in this patch.

The same two species, *Dodonaea viscosa* subsp. *angustifolia* and *Vachellia sieberiana*, also show the highest frequency and RF in Micha, pointing towards a similar species dominance as in Gerado. However, the presence of *Allophylus abyssinicus* with a lower frequency and RF indicates a variation in the understory composition between Micha and Gerado. Similarly, in Mekelet, *Vachellia sieberiana* shows the highest frequency and RF, present in 100% of the plots (S5 File).

#### Basal area.

In Micha, the basal area varied from 2.84 to 0.02 m^2^ ha^-1^; in Mekelet, from 7.25 to 0.01 m^2^ ha^-1^; and in Gerado, from 5.41 to 0.02 m^2^ ha^-1^. The highest basal area in Gerado was recorded for *Olea europaea* L. subsp. *cuspidata*, followed by *Pittosporum viridiflorum* and *Vachellia sieberiana*, indicating their structural dominance in the patch.. In contrast, Micha is characterized by the dominance of *Vachellia sieberiana*, with *Juniperus procera* and *Olea europaea* L. subsp. *cuspidata* also features prominently, and *Euphorbia abyssinica* appears as the least dominant among the top species, suggesting a diversity in species roles within the ecosystem. Similarly, Mekelet’s vegetation structure is governed by the dominance of *Vachellia sieberiana*, with *Olea europaea* L. subsp. *cuspidata* and *Dodonaea viscosa* subsp. *angustifolia* following in BA, and *Juniperus procera* occupying a smaller yet significant ecological niche as one of the top five species.

### Diameter at breast height (DBH) structure of the forest patches

**Fig 5:**
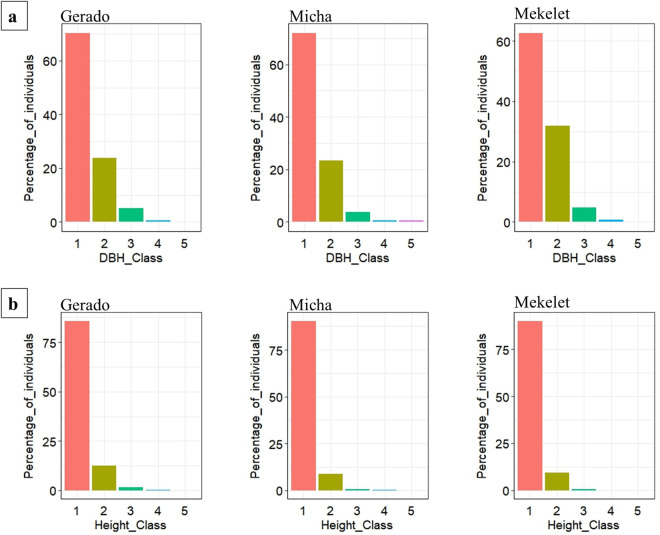
DBH and frequency distribution class. **(a)** Distribution of woody species density among DBH classes (1 = 2.5–9.0 cm, 2 = 9.1–17.0 cm, 3 = 17.1–25.0 cm, 4 = 25.1–32 cm, 5 = 32.1–43.0 cm). **(b)** Frequency distribution by height class of woody species (1 = 2.0–6.0 m, 2 = 6.1–10.0 m, 3 = 10.1–15.0 m, 4 = 15.1–20.0 m, 5 = 20.1–26.0 m).

The DBH distribution revealed that most woody individuals in all three forest patches fall within the smallest size class (2.5–9.0 cm), reflecting high numbers of younger or smaller trees (Fig 5). This pattern suggests ongoing recruitment but may also reflect a lack of larger individuals due to selective cutting. Mekelet, in particular, had a sharper decline in larger DBH classes, consistent with its higher disturbance score.

Micha (71.9%) had the highest proportion of trees in this class, followed closely by Gerado (70.3%) and then Mekelet (62.6%). This indicates a strong presence of younger trees across all sites, with Micha having a slightly more significant proportion of these younger trees. The slightly higher percentage in Micha could indicate either a more diverse range of young species or a more conducive environment for young tree growth.

As the tree size increases, there is a notable decrease in the percentage of trees in all sites. However, Mekelet stands out with a relatively higher percentage (31.9%) in the 9.1–17.0 cm class compared to Gerado (23.8%) and Micha (23.3%). This could imply that Mekelet has a more substantial intermediate-sized tree population. The trend continues in the 17.1–25.0 cm class, though the differences among the sites become less pronounced. In contrast, the lower percentages in Gerado and Micha could imply either a less diverse range of intermediate-sized species or factors limiting their growth. In the larger DBH classes (25.1 cm – 43.0 cm), which indicate mature, large-diameter trees, all sites show minimal percentages. Gerado, Micha, and Mekelet exhibit a similar pattern, suggesting a limited presence of older, larger trees across the sites. This could suggest limited species diversity in the mature tree segment, possibly due to historical logging, anthropogenic factors (cutting, charcoal production, firewood collection), or other ecological factors. The similar patterns across the sites might reflect a regional trend or a shared ecological history.

### Height class of the species in the study site

Across the forest patches of Gerado, Micha, and Mekelet, a pronounced dominance of shorter trees (2.0–6.0 meters) was observed, with respective percentages of 85.82%, 90.47%, and 90.06% (Fig 5). This suggests a youthful or regenerating forest demographic, potentially influenced by a combination of environmental conditions, forest management practices by the local community, or recent growth patterns. While the specific management strategies, if any, employed within these forests remain unclear, it is plausible that practices such as selective logging, controlled burns, or reforestation efforts could contribute to the observed patterns. The subsequent height class (6.1–10.0 meters) shows a substantial decline in tree population, with Gerado at 12.51%, Micha at 8.64%, and Mekelet at 9.29%, reflecting a common ecological trend where fewer trees reach mid-sized maturity.

### Importance value index (IVI)

The Importance Value Index (IVI) provides insight into the ecological dominance of woody species within each forest patch. In Gerado, *Olea europaea* L. subsp. *cuspidata* had the highest IVI (46.95), followed by *Pittosporum viridiflorum* and *Dodonaea viscosa* subsp. *angustifolia*. In Micha, *Dodonaea viscosa* subsp. *angustifolia* ranks as the most important species, with an IVI of 43.23. In Mekelet, *Vachellia sieberiana* had the highest IVI (83.84), followed by *Olea europaea* L. subsp. *cuspidata* and *Dodonaea viscosa* subsp. *angustifolia*, reflecting the dominance of disturbance-tolerant species in this patch. These species play a central role in the community structure of their respective patches and reflect the varying ecological conditions and disturbance levels across sites.

### Population structure of some selected woody plant species

**Fig 6:**
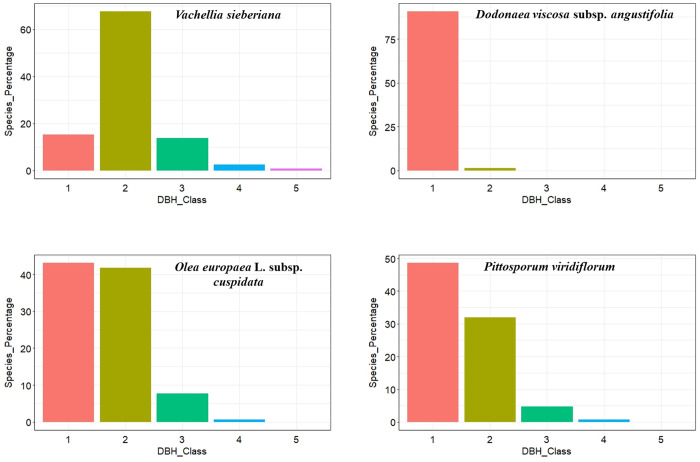
Population structure pattern of selected woody plant species in the study area.

In this study we identified three population structure patterns based on the distribution of diameter at breast height (DBH) classes in selected species (Fig 6). These patterns include the inverted J-shaped pattern, broken inverted J-shaped pattern and bell-shaped pattern. The inverted J-shaped pattern exhibited the highest frequencies in lower size classes and the gradual declining towards higher size classes. Examples of species displaying this pattern include *Olea europaea* L. subsp. *cuspidata* and *Pittosporum viridiflorum.* Broken inverted J-shaped pattern showed the percentage of individuals in the lower DBH class 1 and 2 is very high but becoming lower in the highest DBH classes even nothing in some DBH class 3, 4 and 5. This pattern is manifested by *Dodonaea viscosa* subsp. *angustifolia*. The bell-shaped pattern also showed species where the highest percentages of individuals are in the middle DBH class and smaller percentages in the lower and higher DBH classes. This pattern is manifested by *Vachellia sieberiana*.

## Discussion

The present study recorded a total of 55 woody species across the three forest patches, which falls within the range reported for other Dry Afromontane Forests (DAF) in Ethiopia. However, the richness observed here is slightly lower than in some comparable sites [27]. This variation may be explained by local climate and topographic conditions, environmental heterogeneity, regeneration success, edaphic influences, and the intensity of anthropogenic disturbances [25,41,42]. Rarefaction analyses provided further confirmation that observed differences in woody species richness across forest patches are ecologically meaningful and not artifacts of sampling effort. Both sample-based curves (vegan) and individual-based rarefaction with extrapolation (iNEXT) consistently revealed the same gradient: Gerado > Micha > Mekelet. The Gerado forest maintained the highest richness and species turnover, while Mekelet showed the lowest diversity, plateauing quickly, which likely reflects its higher levels of anthropogenic disturbance. These findings align with prior work indicating that disturbance can severely reduce woody species richness in dry Afromontane forests of Ethiopia [7,43].

Moreover, the consistency of patterns across two rarefaction approaches strengthens confidence in our patch-level comparison framework. Given the relatively low number of individuals per quadrat (mean ± SD = 27.7 ± 6.7; range 13–51), rarefaction at the plot level was limited, but both analyses at the patch and individual levels produced convergent results. Thus, patch-level comparisons remain the most appropriate approach for heterogeneous forest systems, where logistical and ecological constraints often limit plot-based rarefaction [44,45]. For example, Tamiru Lemi et al. (2023) found 42 woody plant species in Dindin Natural Forest, southeast of Ethiopia [46], Metsehet Yinebeb et al. (2023) reported 34 tree species in Melit, Addisinagulit, and Yetegan (Megabzer) forest patches, Gozamin district of Northwest Ethiopia [34], while Yitayih Dagne and Liyew Birhanu (2023) identified 42 tree species in Guard dry Afromontane forest of northwestern Ethiopia [47]. Shemsu Ahmed et al. (2022) also reported 55 woody species in Gennemar dry Afromontane forest in southern Ethiopia [48], Fitsum Temesgen (2021) identified 46 tree species in Kafta Sheraro National Park Dry Forest, Tigray Region, Ethiopia [27], Getie Mucheye et al., (2022) found 59 woody species in Gelawoldie community forest, northwestern Ethiopia [49], Getaneh Gebeyehu et al. (2019) identified 55 woody plant species in dry Afromontane forests of Awi Zone, northwestern Ethiopia [50], Gebremichael Fisaha et al. (2013) reported 62 woody species in Wof Washa natural forest, northeast Ethiopia [51]. The plant family Fabaceae, which turned out to be the most dominant family in the present study, is the first-ranked and the most species-rich family in the Flora of Ethiopia and Eritrea [8,42]. The dominance of Fabaceae in the study sites may be due to the ability of its species to fix nitrogen, disperse effectively, and adapt to diverse ecological and physiological conditions [42,52–54].

The identification of four significantly distinct (*p* ≤ 0.001) community types reflects floristic differentiation, rather than relative diversity, among the studied forest patches. These community types are not strictly confined to individual forest patches, suggesting that species composition is influenced more by ecological conditions than by geographic separation. The MRRP results show that there are significant differences (R = 0.4703, *p* ≤ 0.001) among the four community types. This could be attributed to environmental and anthropogenic factors that cause the variation in species composition among the plant communities [34,42,55,56]. The three forest patches also exhibited a moderate degree of similarity in species composition (Ss Mean ± Sd = 0.65 ± 0.08), which may be attributed to their geographical proximity and the influence of comparable environmental conditions [16]. The RDA results provide further insight into the factors driving this variation. Communities 1 and 2 were associated with lower elevations, while Community 3 was typical of steeper slopes and displayed a broader altitudinal range. Community 4, the most widespread, was strongly correlated with higher levels of tree cutting, suggesting it represents areas under significant anthropogenic pressure.

The fact that communities were distributed across multiple forest patches also indicates that environmental gradients and human disturbances rather than location alone are the key determinants of vegetation structure. This has important implications for conservation planning, underscoring the need to consider ecological function and disturbance intensity across landscapes rather than focusing only on administrative boundaries. Similarly, various researchers in Ethiopia indicated that altitude is a key environmental factor determining the vegetation distribution pattern [34,42,55,57–59].

The dominance of *Dodonaea viscosa* subsp. *angustifolia* in Gerado and Micha is a common finding in DAF of Ethiopia and semi-arid African woodlands and may be linked to its adaptability to drier conditions [60–62]. The prevalence of *Vachellia sieberiana* in Mekelet can be compared to its prominence in East African savannas, often associated with overgrazing protection that favors its growth [63]. In this study, species dominance patterns were more closely aligned with community types and environmental factors than with geographic location alone. In Community Type 1, *Hesperocyparis lusitanica* and *Juniperus procera* was dominant, likely reflecting its adaptation to higher-altitude sites with reduced disturbance. Community Type 2 was characterized by the dominance of *Pittosporum viridiflorum* and *Dodonaea viscosa* subsp. *angustifolia*, species associated with sloped areas and intermediate cutting pressure. In Community Type 3, *Olea europaea* L. subsp. *cuspidata* dominated, consistent with its tolerance to moderate disturbance. Finally, Community Type 4 was dominated by *Vachellia sieberiana*, which is well adapted to sites with higher cutting intensity and anthropogenic pressure. The dominance of *Dodonaea viscosa* subsp. *angustifolia*, *Olea europaea* L. subsp. *cuspidata*, and *Vachellia sieberiana* could be attributed to their drought tolerance and adaptability to disturbed environments [64]. Over all, the identified dominant and important woody plant species in the forest patches are a common finding in DAF of Ethiopia and may be linked to its adaptability to drier conditions [61,62]. In this study, the pattern of species frequency aligns with broader ecological observations across Ethiopia, where specific species exhibit dominance due to their resilience to environmental stresses and disturbances, potentially shaping the forest structure [65, 66].

The study results revealed that the DBH and height classes of the forest patches had inverted J shape distribution patterns attributed to the stable population status [52,67,68]. The study shows that while younger trees dominate in all sites, there are differences in tree and shrub size distribution. Micha tends to have a slightly higher proportion of very young trees, whereas Mekelet shows a tendency for a more substantial presence in the intermediate-size classes. The slightly higher percentage in Micha could indicate either a more diverse range of young species or a more conducive environment for young tree growth. In this study, the scarcity of trees in the tallest categories (10.1–15.0 meters and beyond) across all patches, typically below 2%, suggests a notable absence of mature trees, possibly due to historical harvesting, natural thinning, or other restrictive growth factors. The DBH class distributions, dominated by individuals in the smallest size class, suggest active forest regeneration [69]. However, the near absence of larger trees in Mekelet where tree cutting and agricultural expansion are most intense implies disruption of regeneration before maturation. Such land-use pressures simplify stand structure and shift species composition toward fast-growing, disturbance-tolerant taxa [70]. The IVI value indicated that, *Olea europaea* L*.* subsp. *cuspidata*, *Pittosporum viridiflorum*, *Dodonaea viscosa* subsp. *angustifolia* and *Vachellia sieberiana* are the most important species in the study area. This indicates their dominance and prevalence in terms of density, frequency, and canopy cover within the forest patches.

The population structure pattern of selected woody plant species based on their Importance Value Index (IVI) in the studied forest patches reveals significant ecological insights. *Pittosporum viridiflorum* and *Olea europaea* L. subsp. *cuspidata* exhibit an inverted J-shaped pattern, indicative of healthy regeneration but necessitating further investigation into why fewer individuals reach maturity [40]. This could point towards potential issues in the later stages of growth, such as competition or anthropogenic factors. *Dodonaea viscosa* subsp. *angustifolia* shows a broken inverted J-shaped pattern, emphasizing the adverse effects of selective cutting. This highlights the critical need for implementing sustainable resource management practices to ensure long-term viability [42]. Meanwhile, *Vachellia sieberiana* displays a bell-shaped pattern, suggesting a stable population overall. However, there is a need to focus on the factors that hinder individuals from surviving into the largest diameter at breast height (DBH) classes. Addressing these factors is essential for maintaining the ecological balance and ensuring the persistence of these species within the forest ecosystem [71].

Anthropogenic disturbances including cutting, firewood collection, charcoal production, agricultural expansion, grazing, and browsing are important factors that affect the species distribution and vegetation structure of the forest patches in this study. Many studies indicated that anthropogenic factors often have a pronounced effect on plant species diversity, richness distribution pattern and vegetation structure of forest ecosystems [27,42,47,72,73]. In Gerado, the observed disturbance level was moderate, with cutting and agricultural expansion being the dominant pressures. This may reflect a relatively less intensive or more dispersed pattern of land use compared to Mekelet, where forest degradation was most severe. Field observations suggested that agricultural activities in Gerado occur at some distance from the core forest, reducing direct pressure on woody vegetation. However, these impressions were not supported by quantitative data on land use intensity or population proximity, which we acknowledge as a limitation. The Mekelet forest patch faces the highest disturbance, primarily driven by intense cutting and firewood collection. This heightened impact may be influenced by factors such as higher population density, increased demand for resources, or limited alternative livelihood options. Among the anthropogenic factors, charcoal production has varied impacts across forest ecosystems in Ethiopia [74,75]. Charcoal production exhibits diverse impacts across the three forest patches, with Gerado experiencing minimal influence, possibly due to a strong local commitment to protect the forest. In the Micha forest patch, an even lower impact was observed, suggesting conservation efforts, cultural norms, and local initiatives play a role in discouraging or regulating charcoal production. These protective actions by the local community in Gerado and Micha contribute to the preservation of their respective forest patches. In contrast, the Mekelet forest patch shows a moderate impact from charcoal production, potentially driven by factors such as increased population pressure and reliance on charcoal as alternative energy sources.

These findings align with studies in other dry Afromontane forests such as Hugumbirda-Gratkhassu and Gennemar, where anthropogenic pressures were also primary drivers of floristic simplification [39,59]. However, anthropogenic pressures such as agricultural expansion, overgrazing, and population growth have been primary drivers influencing the floristic composition and structural dynamics of these forests. Excessive cutting of ecologically and economically important tree species for construction and other uses has further altered species composition and regeneration patterns, pushing some forest patches into early secondary stages of development [48]. Environmental factors like altitude, slope, and aspect also interact with these anthropogenic impacts to shape plant community distribution, but human-induced disturbances remain dominant in determining the current floristic structure of Ethiopia’s dry Afromontane forests [76,77].

By integrating vegetation structure with disturbance analysis, the study provides novel empirical evidence that slope and human impact, in addition to altitude, significantly influence community composition in dry Afromontane forests. This nuanced understanding enhances current ecological models of vegetation distribution in Ethiopia and supports the design of more effective, site-specific conservation strategies. Restoration and management programs should prioritize areas like Community 4 zones, where anthropogenic pressure is highest and ecological degradation most advanced. This study contributes to the existing literature by combining floristic analysis with environmental and anthropogenic disturbance assessments to explain community structure and species distribution patterns. While previous studies primarily emphasized altitude and soil conditions, our findings highlight the additional importance of slope and human impact, especially cutting and agricultural expansion, in shaping woody species composition.

## Conclusion

This study assessed woody species composition, community structure, and the influence of environmental and anthropogenic factors in three dry Afromontane forest patches of North Wollo, Ethiopia. A total of 55 species were recorded, grouped into four statistically distinct communities shaped by slope, altitude, and cutting pressure. Species richness was highest in Gerado and lowest in Mekelet, where structural degradation reflected intensive human disturbance. Ecologically dominant species included *Dodonaea viscosa* subsp. *angustifolia*, *Olea europaea* L. subsp. *cuspidata*, and *Vachellia sieberiana*. These findings underscore the urgent need for integrated forest management and conservation strategies that balance ecological sustainability with socio-economic considerations. To mitigate ongoing degradation, we recommend implementing targeted policy measures to regulate tree cutting and curb charcoal production, particularly in severely affected areas. Concurrently, promoting sustainable land-use practices such as agroforestry systems and energy-efficient technologies can reduce dependence on forest resources while supporting livelihoods. Strengthening collaboration with local communities is critical; this includes reinforcing traditional conservation practices (e.g., seasonal access restrictions and informal protection zones) and integrating them into formal frameworks through participatory forest management (PFM). Future research should incorporate socio-demographic and spatial land use data to better understand human-forest interactions and to inform more effective, inclusive, and scalable conservation models.

## Supporting information

S1 File Vegetation data collected from Gerado, Micha and Mekelet forest patches. (CSV)

S2 File Environmental data. (CSV)

S3 File List of woody plant species recorded from Gerado, Micha and Mekelet forest patches, North Wollo Zone, Ethiopia. (DOCX)

S4 File Woody species list in Gerado forest patch with their respective Density (D), Frequency (F), Basal Area (BA), Relative Density (RD), Relative Frequency (RF), Relative Dominance (RDO), and Importance Value Index (IVI). (DOCX)

S5 File Synoptic cover abundance value of species in each community type. (DOCX)

S6 File Density, frequency, basal area, dominance, and importance value index of top five woody plant species across the three forest patches. (DOCX)

S7 File Community type classification. (DOCX)

S8 File Optimal number of clusters in the studied forest patches. (DOCX)

S9 File R Code and results for rarefaction and extrapolation analysis. (DOCX)
