# Supplementary material for: Influence of environmental and anthropogenic factors on forest patch composition and structure in North Wollo Zone, Amhara region, Ethiopia
Source: PLoS One. 2025 Sep 23;20(9):e0332831. doi: 10.1371/journal.pone.0332831 (PMC12456791; doi:10.1371/journal.pone.0332831)
Supplement: S3 File — (DOCX) [file pone.0332831.s003.docx]

**S3 File: List of woody plant species recorded from Gerado, Micha and Mekelet forest patches, North Wollo Zone, Ethiopia**

| **Species name** | **Family** | **Local name** | **Growth form (GF)** | **Occurrence in the forest patches** |
| --- | --- | --- | --- | --- |
| *Acacia decurrens* (J.C.Wendl.) Willd. | Fabaceae | Akacha | T | 1 |
| *Afrocarpus falcatus* (Thunb.) C.N.Page | Podocarpaceae | Zigba | T | 1 |
| *Albizia gummifera* (J.F.Gmel.) C.A.Sm. | Fabaceae | Sesa | T | 1 |
| *Allophylus abyssinicus* (Hochst.) Radlk. | Sapindaceae | Embis | T | 1, 2, 3 |
| *Arundo donax* L. | Poaceae | Shenbeko | S | 1 |
| *Calotropis procera* (Aiton) W.T.Aiton | Apocynaceae | Tobia | S | 1 |
| *Calpurnia aurea* (Aiton) Benth. | Fabaceae | Digita | S | 1 |
| *Carissa spinarum* L. | Apocynaceae | Agam | S | 1, 2, 3 |
| *Casuarina equisetifolia* L. | Casuarinaceae | Shiwshiwa | T | 1 |
| *Celtis africana* Burm.f. | Cannabaceae | Awrarisie | S | 1 |
| *Citrus aurantiifolia* (Christm.) Swingle | Rutaceae | Lomi | S | 1 |
| *Cordia africana* Lam. | Boraginaceae | Wanza | T | 1 |
| *Croton macrostachyus* Hochst. ex Delile | Euphorbiaceae | Mekanisa | T | 2, 3 |
| *Dichrostachys cinerea* (L.) Wight & Arn. | Fabaceae | Gorgoro/Ader | S | 2 |
| *Dodonaea viscosa* subsp. angustifolia (L.f.) J.G.West | Sapindaceae | Kitkita | S | 1, 2, 3 |
| *Dombeya torrida* (J.F.Gmel.) Bamps | Malvaceae | Danisa/Kuncho | S | 1,2 |
| *Ehretia cymosa* Thonn | Boraginaceae | Ulaga | T | 1 |
| *Eucalyptus camaldulensis* Dehnh. | Myrtaceae | Key Bahirzaf | T | 1, 2, 3 |
| *Euclea racemosa* L. | Ebenaceae | Dedeho | S | 1, 2, 3 |
| *Euphorbia abyssinica* J.F.Gmel. | Euphorbiaceae | Qulqual | S | 1, 2, 3 |
| *Euphorbia tirucalli* L. | Euphorbiaceae | Kinchibt | S | 1, 2 |
| *Faidherbia albida* (Delile) A.Chev. | Fabaceae | Gerbi | T | 1, 3 |
| *Ficus capreifolia* Delile | Moraceae | Beles | T | 2 |
| *Ficus sur* Forssk. | Moraceae | Shola | T | 2 |
| *Ficus vasta* Forssk. | Moraceae | Warka | T | 1 |
| *Galiniera saxifraga* (Hochst.) Bridson | Rubiaceae | Buna Mesay | S | 1 |
| *Grevillea robusta* A.Cunn. ex R.Br. | Proteaceae | Gravilea | T | 1 |
| *Grewia bicolor* Juss. | Malvaceae | Sefa | S | 1 |
| *Grewia ferruginea* Hochst. ex A.Rich. | Malvaceae | Lenquata | S | 1, 2, 3 |
| *Gymnanthemum amygdalinum* (Delile) Sch.Bip. | Asteraceae | Girawa | S | 1, 2, 3 |
| *Gymnosporia senegalensis* (Lam.) Loes. | Celastraceae | Atat | S | 1, 2, 3 |
| *Hesperocyparis lusitanica* (Mill.) Bartel | Cupressaceae | Yeferenj Tsid | T | 1, 2 |
| *Heteromorpha arborescens* (Spreng.) Cham. & Schltdl. | Apiaceae | Yejib Mirkuz | S | 1, 2 |
| *Jacaranda mimosifolia* D.Don | Bignoniaceae | Yetebenja Zaf /Jakaranda | T | 1 |
| *Juniperus procera* Hochst. ex Endl. | Cupressaceae | Yehabesha Tsid | T | 1, 2, 3 |
| *Moringa oleifera* Lam. | Moringaceae | Shiferaw/Moringa | T | 1 |
| *Myrsine africana* L. | Primulaceae | Kechem | S | 1, 2, 3 |
| *Olea europaea* L. subsp. *cuspidata* (Wall. & G.Don) Cif. | Oleaceae | Woyra | T | 1, 2, 3 |
| *Opuntia ficus-indica* (L.) Mill. | Cactaceae | Qulqual / Manka/Beles/Wedi | S | 1 |
| *Osyris lanceolata* Hochst. & Steud. | Santalaceae | Keret | S | 1, 2, 3 |
| *Pittosporum viridiflorum* Sims | Pittosporaceae | Kefeto | T | 1, 2, 3 |
| *Premna schimperi* Engl. | Lamiaceae | Checho | S | 1, 3 |
| *Pterolobium stellatum* (Forssk.) Brenan | Fabaceae | Kentafa | S | 1, 2, 3 |
| *Rhamnus prinoides* L'Hér. | Rhamnaceae | Gesho | S | 1 |
| *Rosa abyssinica* R.Br. ex Lindl. | Rosaceae | Kega | S | 1, 2 |
| *Schinus molle* L. | Anacardiaceae | Kundo-berberie | T | 1, 2 |
| *Searsia glutinosa* (Hochst. ex A.Rich.) Moffett | Anacardiaceae | Embis/2 | S | 1, 2 |
| *Searsia retinorrhoea* (Steud. ex Oliv.) Moffett | Anacardiaceae | Talo | S | 1, 2, 3 |
| *Senegalia brevispica* (Harms) Seigler & Ebinger | Fabaceae | Kontr | S | 1, 2 |
| *Vachellia amythethophylla* (Steud. ex A.Rich.) Kyal. & Boatwr. | Fabaceae | Lafto | T | 1 |
| *Vachellia etbaica* (Schweinf.) Kyal. & Boatwr. | Fabaceae | Tikur Girar | T | 1, 2, 3 |
| *Vachellia seyal* (Delile) P.J.H.Hurter | Fabaceae | Wacho Girar | T | 2 |
| *Vachellia sieberiana* (DC.) Kyal. & Boatwr. | Fabaceae | Key Girar | T | 1, 2, 3 |
| *Vachellia tortilis* (Forssk.) Galasso & Banfi | Fabaceae | Deweni Grar, Korera | T | 1 |
| *Ziziphus spina-christi* (L.) Desf. | Rhamnaceae | Kurkura, Geba | T | 1, 2 |

Key: Growth form, GF (Tree, T; Shrub, S). Forest patch, FP (1, Gerado forest patch; 2, Micha forest patch; 3, Mekelet forest patch)
