# Supplementary material for: Influence of environmental and anthropogenic factors on forest patch composition and structure in North Wollo Zone, Amhara region, Ethiopia
Source: PLoS One. 2025 Sep 23;20(9):e0332831. doi: 10.1371/journal.pone.0332831 (PMC12456791; doi:10.1371/journal.pone.0332831)
Supplement: S4 File — (DOCX) [file pone.0332831.s004.docx]

**S4 File: Woody species list in Gerado forest patch with their respective Density (D), Frequency (F), Basal Area (BA), Relative Density (RD), Relative Frequency (RF), Relative Dominance (RDO), and Importance Value Index (IVI)**

| **No.** | **Species name** | **D (h^-1^)** | **F** | **BA (m^2^h^-1^)** | **RD** | **RF** | **RDO** | **IVI** |
| --- | --- | --- | --- | --- | --- | --- | --- | --- |
| 1 | *Acacia decurrens* (J.C.Wendl.) Willd. | 6.40 | 11.63 | 0.18 | 0.28 | 1.24 | 0.69 | 2.21 |
| 2 | *Afrocarpus falcatus* (Thunb.) C.N.Page | 2.91 | 4.65 | 0.05 | 0.13 | 0.50 | 0.19 | 0.82 |
| 3 | *Albizia gummifera* (J.F.Gmel.) C.A.Sm. | 1.74 | 2.33 | 0.00 | 0.08 | 0.25 | 0.01 | 0.33 |
| 4 | *Allophylus abyssinicus* (Hochst.) Radlk. | 30.81 | 32.56 | 0.08 | 1.34 | 3.47 | 0.31 | 5.12 |
| 5 | *Arundo donax* L. | 4.65 | 2.33 | 0.00 | 0.20 | 0.25 | 0.02 | 0.47 |
| 6 | *Calotropis procera* (Aiton) W.T.Aiton | 5.81 | 4.65 | 0.00 | 0.25 | 0.50 | 0.02 | 0.77 |
| 7 | *Calpurnia aurea* (Aiton) Benth. | 3.49 | 4.65 | 0.01 | 0.15 | 0.50 | 0.04 | 0.69 |
| 8 | *Carissa spinarum* L. | 109.30 | 46.51 | 0.28 | 4.75 | 4.96 | 1.06 | 10.77 |
| 9 | *Casuarina equisetifolia* L. | 1.16 | 2.33 | 0.02 | 0.05 | 0.25 | 0.08 | 0.38 |
| 10 | *Celtis africana* Burm.f. | 6.98 | 9.30 | 0.06 | 0.30 | 0.99 | 0.25 | 1.54 |
| 11 | *Citrus aurantiifolia* (Christm.) Swingle | 1.74 | 2.33 | 0.01 | 0.08 | 0.25 | 0.02 | 0.35 |
| 12 | *Cordia africana* Lam. | 0.58 | 2.33 | 0.10 | 0.03 | 0.25 | 0.37 | 0.64 |
| 13 | *Dodonaea viscosa* subsp. *angustifolia* (L.f.) J.G.West | 519.19 | 95.35 | 1.28 | 22.57 | 10.17 | 4.91 | 37.65 |
| 14 | *Dombeya torrida* (J.F.Gmel.) Bamps | 5.81 | 6.98 | 0.02 | 0.25 | 0.74 | 0.06 | 1.06 |
| 15 | *Ehretia cymosa* Thonn | 6.40 | 4.65 | 0.07 | 0.28 | 0.50 | 0.29 | 1.06 |
| 16 | *Eucalyptus camaldulensis* Dehnh. | 81.98 | 30.23 | 0.93 | 3.56 | 3.23 | 3.57 | 10.36 |
| 17 | *Euclea racemosa* L. | 141.28 | 79.07 | 0.32 | 6.14 | 8.44 | 1.23 | 15.80 |
| 18 | *Euphorbia abyssinica* J.F.Gmel. | 66.28 | 18.60 | 0.90 | 2.88 | 1.99 | 3.46 | 8.32 |
| 19 | *Euphorbia tirucalli* L. | 7.56 | 2.33 | 0.02 | 0.33 | 0.25 | 0.09 | 0.67 |
| 20 | *Faidherbia albida* (Delile) A.Chev. | 2.91 | 4.65 | 0.01 | 0.13 | 0.50 | 0.03 | 0.65 |
| 21 | *Ficus vasta* Forssk. | 0.58 | 2.33 | 0.11 | 0.03 | 0.25 | 0.44 | 0.71 |
| 22 | *Galiniera saxifraga* (Hochst.) Bridson | 2.91 | 4.65 | 0.00 | 0.13 | 0.50 | 0.01 | 0.64 |
| 23 | *Grevillea robusta* A.Cunn. ex R.Br. | 1.74 | 2.33 | 0.10 | 0.08 | 0.25 | 0.38 | 0.70 |
| 24 | *Grewia bicolor* Juss. | 1.16 | 2.33 | 0.00 | 0.05 | 0.25 | 0.02 | 0.31 |
| 25 | *Grewia ferruginea* Hochst. ex A.Rich. | 8.14 | 13.95 | 0.02 | 0.35 | 1.49 | 0.06 | 1.91 |
| 26 | *Gymnanthemum amygdalinum* (Delile) Sch.Bip. | 5.23 | 4.65 | 0.01 | 0.23 | 0.50 | 0.05 | 0.77 |
| 27 | *Gymnosporia senegalensis* (Lam.) Loes. | 8.72 | 16.28 | 0.02 | 0.38 | 1.74 | 0.09 | 2.20 |
| 28 | *Hesperocyparis lusitanica* (Mill.) Bartel | 5.81 | 9.30 | 0.18 | 0.25 | 0.99 | 0.71 | 1.95 |
| 29 | *Heteromorpha arborescens* (Spreng.) Cham. & Schltdl. | 13.95 | 9.30 | 0.06 | 0.61 | 0.99 | 0.25 | 1.85 |
| 30 | *Jacaranda mimosifolia* D.Don | 2.91 | 2.33 | 0.06 | 0.13 | 0.25 | 0.23 | 0.60 |
| 31 | *Juniperus procera* Hochst. ex Endl. | 93.02 | 27.91 | 4.30 | 4.04 | 2.98 | 16.56 | 23.58 |
| 32 | *Moringa oleifera* Lam. | 2.91 | 2.33 | 0.02 | 0.13 | 0.25 | 0.09 | 0.46 |
| 33 | *Myrsine africana* L. | 62.21 | 27.91 | 0.09 | 2.70 | 2.98 | 0.35 | 6.03 |
| 34 | *Olea europaea* L. subsp. *cuspidata* (Wall. & G.Don) Cif. | 394.77 | 83.72 | 5.42 | 17.16 | 8.93 | 20.86 | 46.95 |
| 35 | *Opuntia ficus-indica* (L.) Mill. | 1.16 | 2.33 | 0.03 | 0.05 | 0.25 | 0.10 | 0.40 |
| 36 | *Osyris lanceolata* Hochst. & Steud. | 45.93 | 37.21 | 0.12 | 2.00 | 3.97 | 0.45 | 6.41 |
| 37 | *Pittosporum viridiflorum* Sims | 275.00 | 90.70 | 4.96 | 11.95 | 9.68 | 19.08 | 40.71 |
| 38 | *Premna schimperi* Engl. | 5.81 | 2.33 | 0.01 | 0.25 | 0.25 | 0.03 | 0.53 |
| 39 | *Pterolobium stellatum* (Forssk.) Brenan | 24.42 | 23.26 | 0.07 | 1.06 | 2.48 | 0.27 | 3.81 |
| 40 | *Rhamnus prinoides* L'Hér. | 1.74 | 2.33 | 0.00 | 0.08 | 0.25 | 0.01 | 0.33 |
| 41 | *Rosa abyssinica* R.Br. ex Lindl. | 1.74 | 2.33 | 0.00 | 0.08 | 0.25 | 0.01 | 0.33 |
| 42 | *Schinus molle* L. | 2.33 | 2.33 | 0.02 | 0.10 | 0.25 | 0.08 | 0.43 |
| 43 | *Searsia glutinosa* (Hochst. ex A.Rich.) Moffett | 27.91 | 13.95 | 0.10 | 1.21 | 1.49 | 0.39 | 3.09 |
| 44 | *Searsia retinorrhoea* (Steud. ex Oliv.) Moffett | 111.63 | 48.84 | 0.41 | 4.85 | 5.21 | 1.57 | 11.63 |
| 45 | *Senegalia brevispica* (Harms) Seigler & Ebinger | 4.65 | 4.65 | 0.01 | 0.20 | 0.50 | 0.03 | 0.73 |
| 46 | *Vachellia amythethophylla* (Steud. ex A.Rich.) Kyal. & Boatwr. | 4.65 | 4.65 | 0.05 | 0.20 | 0.50 | 0.21 | 0.91 |
| 47 | *Vachellia etbaica* (Schweinf.) Kyal. & Boatwr. | 23.84 | 23.26 | 0.51 | 1.04 | 2.48 | 1.98 | 5.50 |
| 48 | *Vachellia sieberiana* (DC.) Kyal. & Boatwr. | 152.91 | 95.35 | 4.86 | 6.65 | 10.17 | 18.70 | 35.52 |
| 49 | *Vachellia tortilis* (Forssk.) Galasso & Banfi | 2.91 | 2.33 | 0.06 | 0.13 | 0.25 | 0.22 | 0.60 |
| 50 | *Ziziphus spina-christi* (L.) Desf. | 2.91 | 4.65 | 0.03 | 0.13 | 0.50 | 0.10 | 0.72 |

Woody species list in Micha forest patch with their respective Density (D), Frequency (F), Basal Area (BA), Relative Density (RD), Relative Frequency (RF), Relative Dominance (RDO), and Importance Value Index (IVI)

| **No.** | **Species name** | **D (h^-1^)** | **F** | **BA (m^2^h^-1^)** | **RD** | **RF** | **RDO** | **IVI** |
| --- | --- | --- | --- | --- | --- | --- | --- | --- |
| 1 | *Allophylus abyssinicus* (Hochst.) Radlk. | 84.82 | 57.14 | 0.19 | 4.31 | 6.72 | 1.43 | 12.46 |
| 2 | *Carissa spinarum* L. | 28.57 | 21.43 | 0.06 | 1.45 | 2.52 | 0.42 | 4.39 |
| 3 | *Croton macrostachyus* Hochst. ex Delile | 3.57 | 3.57 | 0.02 | 0.18 | 0.42 | 0.12 | 0.72 |
| 4 | *Dichrostachys cinerea* (L.) Wight & Arn. | 2.68 | 3.57 | 0.01 | 0.14 | 0.42 | 0.09 | 0.64 |
| 5 | *Dodonaea viscosa* subsp. *angustifolia* (L.f.) J.G.West | 548.21 | 85.71 | 0.72 | 27.83 | 10.08 | 5.31 | 43.23 |
| 6 | *Dombeya torrida* (J.F.Gmel.) Bamps | 52.68 | 10.71 | 0.12 | 2.67 | 1.26 | 0.92 | 4.85 |
| 7 | *Eucalyptus camaldulensis* Dehnh. | 92.86 | 46.43 | 1.85 | 4.71 | 5.46 | 13.66 | 23.83 |
| 8 | *Euclea racemosa* L. | 57.14 | 42.86 | 0.10 | 2.90 | 5.04 | 0.75 | 8.69 |
| 9 | *Euphorbia abyssinica* J.F.Gmel. | 70.54 | 32.14 | 1.05 | 3.58 | 3.78 | 7.74 | 15.10 |
| 10 | *Euphorbia tirucalli* L. | 6.25 | 3.57 | 0.02 | 0.32 | 0.42 | 0.16 | 0.90 |
| 11 | *Ficus capreifolia* Delile | 1.79 | 3.57 | 0.01 | 0.09 | 0.42 | 0.04 | 0.55 |
| 12 | *Ficus sur* Forssk. | 0.89 | 3.57 | 0.00 | 0.05 | 0.42 | 0.01 | 0.48 |
| 13 | *Grewia ferruginea* Hochst. ex A.Rich. | 11.61 | 7.14 | 0.02 | 0.59 | 0.84 | 0.18 | 1.61 |
| 14 | *Gymnanthemum amygdalinum* (Delile) Sch.Bip. | 0.89 | 3.57 | 0.00 | 0.05 | 0.42 | 0.02 | 0.48 |
| 15 | *Gymnosporia senegalensis* (Lam.) Loes. | 34.82 | 32.14 | 0.06 | 1.77 | 3.78 | 0.41 | 5.96 |
| 16 | *Hesperocyparis lusitanica* (Mill.) Bartel | 27.68 | 28.57 | 0.53 | 1.41 | 3.36 | 3.90 | 8.67 |
| 17 | *Heteromorpha arborescens* (Spreng.) Cham. & Schltdl. | 11.61 | 3.57 | 0.03 | 0.59 | 0.42 | 0.25 | 1.26 |
| 18 | *Juniperus procera* Hochst. ex Endl. | 117.86 | 53.57 | 2.25 | 5.98 | 6.30 | 16.63 | 28.92 |
| 19 | *Myrsine africana* L. | 79.46 | 25.00 | 0.07 | 4.03 | 2.94 | 0.49 | 7.46 |
| 20 | *Olea europaea* L. subsp. *cuspidata* (Wall. & G.Don) Cif. | 216.96 | 82.14 | 2.08 | 11.02 | 9.66 | 15.37 | 36.05 |
| 21 | *Osyris lanceolata* Hochst. & Steud. | 50.00 | 32.14 | 0.10 | 2.54 | 3.78 | 0.75 | 7.07 |
| 22 | *Pittosporum viridiflorum* Sims | 121.43 | 78.57 | 0.96 | 6.17 | 9.24 | 7.08 | 22.49 |
| 23 | *Pterolobium stellatum* (Forssk.) Brenan | 5.36 | 3.57 | 0.01 | 0.27 | 0.42 | 0.05 | 0.74 |
| 24 | *Rosa abyssinica* R.Br. ex Lindl. | 3.57 | 3.57 | 0.00 | 0.18 | 0.42 | 0.02 | 0.62 |
| 25 | *Schinus molle* L. | 1.79 | 3.57 | 0.01 | 0.09 | 0.42 | 0.04 | 0.55 |
| 26 | *Searsia glutinosa* (Hochst. ex A.Rich.) Moffett | 25.89 | 21.43 | 0.05 | 3.49 | 2.52 | 0.36 | 6.38 |
| 27 | *Searsia retinorrhoea* (Steud. ex Oliv.) Moffett | 57.14 | 35.71 | 0.07 | 2.90 | 4.20 | 0.51 | 7.61 |
| 28 | *Senegalia brevispica* (Harms) Seigler & Ebinger | 1.79 | 3.57 | 0.00 | 0.09 | 0.42 | 0.01 | 0.53 |
| 29 | *Vachellia etbaica* (Schweinf.) Kyal. & Boatwr. | 56.25 | 10.71 | 0.19 | 2.86 | 1.26 | 1.42 | 5.54 |
| 30 | *Vachellia seyal* (Delile) P.J.H.Hurter | 15.18 | 17.86 | 0.10 | 0.77 | 2.10 | 0.77 | 3.64 |
| 31 | *Vachellia sieberiana* (DC.) Kyal. & Boatwr. | 135.71 | 85.71 | 2.84 | 6.89 | 10.08 | 20.97 | 37.95 |
| 32 | *Ziziphus spina-christi* (L.) Desf. | 1.79 | 3.57 | 0.02 | 0.09 | 0.42 | 0.12 | 0.63 |

Woody species list in Mekelet forest patch with their respective Density (D), Frequency (F), Basal Area (BA), Relative Density (RD), Relative Frequency (RF), Relative Dominance (RDO), and Importance Value Index (IVI)

| **No.** | **Species name** | **D (h^-1^)** | **F** | **BA (m^2^h^-1^)** | **RD** | **RF** | **RDO** | **IVI** |
| --- | --- | --- | --- | --- | --- | --- | --- | --- |
| 1 | *Allophylus abyssinicus* (Hochst.) Radlk. | 47.92 | 54.17 | 0.12 | 2.27 | 8.18 | 0.75 | 11.20 |
| 2 | *Carissa spinarum* L. | 44.79 | 25.00 | 0.17 | 2.13 | 3.77 | 1.08 | 6.98 |
| 3 | *Croton macrostachyus* Hochst. ex Delile | 6.25 | 12.50 | 0.01 | 0.30 | 1.89 | 0.08 | 2.27 |
| 4 | *Dodonaea viscosa* subsp. *angustifolia* (L.f.) J.G.West | 833.33 | 91.67 | 1.79 | 39.55 | 13.84 | 11.59 | 64.97 |
| 5 | *Eucalyptus camaldulensis* Dehnh. | 141.67 | 50.00 | 1.21 | 6.72 | 7.55 | 7.86 | 22.13 |
| 6 | *Euclea racemosa* L. | 14.58 | 12.50 | 0.01 | 0.69 | 1.89 | 0.07 | 2.65 |
| 7 | *Euphorbia abyssinica* J.F.Gmel. | 17.71 | 12.50 | 0.06 | 0.84 | 1.89 | 0.36 | 3.09 |
| 8 | *Faidherbia albida* (Delile) A.Chev. | 2.08 | 4.17 | 0.00 | 0.10 | 0.63 | 0.02 | 0.75 |
| 9 | *Grewia ferruginea* Hochst. ex A.Rich. | 15.63 | 16.67 | 0.02 | 0.74 | 2.52 | 0.12 | 3.37 |
| 10 | *Gymnanthemum amygdalinum* (Delile) Sch.Bip. | 2.08 | 4.17 | 0.00 | 0.10 | 0.63 | 0.02 | 0.75 |
| 11 | *Gymnosporia senegalensis* (Lam.) Loes. | 26.04 | 25.00 | 0.06 | 1.24 | 3.77 | 0.36 | 5.37 |
| 12 | *Juniperus procera* Hochst. ex Endl. | 17.71 | 20.83 | 0.44 | 0.84 | 3.14 | 2.82 | 6.81 |
| 13 | *Myrsine africana* L. | 56.25 | 20.83 | 0.25 | 2.67 | 3.14 | 1.61 | 7.42 |
| 14 | *Olea europaea* L. subsp. *cuspidata* (Wall. & G.Don) Cif. | 304.17 | 83.33 | 3.57 | 14.43 | 12.58 | 23.12 | 50.14 |
| 15 | *Osyris lanceolata* Hochst. & Steud. | 59.38 | 41.67 | 0.11 | 2.82 | 6.29 | 0.74 | 9.85 |
| 16 | *Pittosporum viridiflorum* Sims | 15.63 | 29.17 | 0.18 | 0.74 | 4.40 | 1.18 | 6.32 |
| 17 | *Premna schimperi* Engl. | 11.46 | 12.50 | 0.06 | 0.54 | 1.89 | 0.41 | 2.84 |
| 18 | *Pterolobium stellatum* (Forssk.) Brenan | 3.13 | 4.17 | 0.01 | 0.15 | 0.63 | 0.04 | 0.82 |
| 19 | *Searsia retinorrhoea* (Steud. ex Oliv.) Moffett | 15.63 | 16.67 | 0.02 | 0.74 | 2.52 | 0.14 | 3.40 |
| 20 | *Vachellia etbaica* (Schweinf.) Kyal. & Boatwr. | 12.50 | 25.00 | 0.11 | 0.59 | 3.77 | 0.69 | 5.05 |
| 21 | *Vachellia sieberiana* (DC.) Kyal. & Boatwr. | 459.38 | 100.00 | 7.25 | 21.80 | 15.09 | 46.95 | 83.84 |
