# Supplementary material for: Influence of environmental and anthropogenic factors on forest patch composition and structure in North Wollo Zone, Amhara region, Ethiopia
Source: PLoS One. 2025 Sep 23;20(9):e0332831. doi: 10.1371/journal.pone.0332831 (PMC12456791; doi:10.1371/journal.pone.0332831)
Supplement: S5 File — (DOCX) [file pone.0332831.s005.docx]

**S6: Density, frequency, basal area, dominance, and importance value index of top five woody plant species across the three forest patches**

a) Density and relative density of top 5 woody plant species across the three forest patches

| **Patch name** | **Species name** | **No. of individuals** | **D** | **Total No. of stems** | **RD** | **Rank** |
| --- | --- | --- | --- | --- | --- | --- |
| **Gerado** | *Dodonaea viscosa* subsp*. angustifolia* | 893 | 519.19 | 3957 | 22.6 | 1 |
|  | *Olea europaea* L. subsp. *cuspidata* | 679 | 394.77 |  | 17.2 | 2 |
|  | *Pittosporum viridiflorum* | 473 | 275.00 |  | 12.0 | 3 |
|  | *Vachellia sieberiana* | 263 | 152.91 |  | 6.6 | 4 |
|  | *Euclea racemosa* | 243 | 141.28 |  | 6.1 | 5 |
| **Micha** | *Dodonaea viscosa* subsp. *angustifolia* | 614 | 548.21 | 2158 | 28.5 | 1 |
|  | *Olea europaea* L. subsp. *cuspidata* | 243 | 216.96 |  | 11.3 | 2 |
|  | *Vachellia sieberiana* | 152 | 135.71 |  | 7.0 | 3 |
|  | *Pittosporum viridiflorum* | 136 | 121.43 |  | 6.3 | 4 |
|  | *Juniperus procera* | 132 | 117.86 |  | 6.1 | 5 |
| **Mekelet** | *Dodonaea viscosa* subsp. *angustifolia* | 800 | 833.333 | 2023 | 39.5 | 1 |
|  | *Vachellia sieberiana* | 441 | 459.375 |  | 21.8 | 2 |
|  | *Olea europaea* L. subsp. *cuspidata* | 292 | 304.167 |  | 14.4 | 3 |
|  | *Eucalyptus camaldulensis* | 136 | 141.667 |  | 6.7 | 4 |
|  | *Osyris lanceolata* | 57 | 59.375 |  | 2.8 | 5 |

b) Frequency and relative frequency of top 5 woody plant species across the three forest patches

| **Patch name** | **Species name** | **No. of individuals** | | **No of plots species occurs** | | **Frequency (%)** | | | **∑ of F of all spp.** | | **RF** | | **Rank** |
| --- | --- | --- | --- | --- | --- | --- | --- | --- | --- | --- | --- | --- | --- |
| Gerado | *Dodonaea viscosa* subsp. *angustifolia* | | 893 | | 41 | | 95.35 | 937.209 | | 10.17 | | 1 | |
|  | *Vachellia sieberiana* | | 263 | | 41 | | 95.35 |  |  | 10.17 | | 1 | |
|  | *Pittosporum viridiflorum* | | 473 | | 39 | | 90.70 |  |  | 9.68 | | 3 | |
|  | *Olea europaea* L. subsp. *cuspidata* | | 679 | | 36 | | 83.72 |  |  | 8.93 | | 4 | |
|  | *Euclea racemosa* | | 243 | | 34 | | 79.07 |  |  | 8.44 | | 5 | |
| Micha | *Dodonaea viscosa* subsp. *angustifolia* | | 614 | | 24 | | 85.71 | 850 | | 10.08 | | 1 | |
|  | *Vachellia sieberiana* | | 152 | | 24 | | 85.71 |  |  | 10.08 | | 1 | |
|  | *Olea europaea* L. subsp. *cuspidata* | | 243 | | 23 | | 82.14 |  |  | 9.66 | | 3 | |
|  | *Pittosporum viridiflorum Sims* | | 136 | | 22 | | 78.57 |  |  | 9.24 | | 4 | |
|  | *Allophylus abyssinicus* | | 95 | | 16 | | 57.14 |  |  | 6.72 | | 5 | |
| Mekelet | *Vachellia sieberiana* | | 441 | | 24 | | 100 | 662.5 | | 15.09 | | 1 | |
|  | *Dodonaea viscosa* subsp*. angustifolia* | | 800 | | 22 | | 91.67 |  |  | 13.84 | | 2 | |
|  | *Olea europaea* L. subsp. *cuspidata* | | 292 | | 20 | | 83.33 |  |  | 12.58 | | 3 | |
|  | *Allophylus abyssinicus* | | 46 | | 13 | | 54.17 |  |  | 8.18 | | 4 | |
|  | *Eucalyptus camaldulensis* | | 136 | | 12 | | 50 |  |  | 7.55 | | 5 | |

c) Basal Area (BA) and dominance of top five woody plant species in Gerado, Micha and Mekelet forest patches

| **Patch name** | **Scientific Name** | **T BA of spp** | **BA (m^2^h^-1^)** | **Dominance** | **∑ of all BA** | **∑ of all BA** | **RDO** | **Rank** |
| --- | --- | --- | --- | --- | --- | --- | --- | --- |
| Gerado | *Olea europaea* L. subsp. *cuspidata* | 54174.318 | 5.417 | 31496.70 | 259763.73 | 25.98 | 20.855 | 1 |
|  | *Pittosporum viridiflorum* | 49564.500 | 4.956 | 28816.57 |  |  | 19.081 | 2 |
|  | *Vachellia sieberiana* | 48566.568 | 4.857 | 28236.38 |  |  | 18.696 | 3 |
|  | *Juniperus procera* | 43025.159 | 4.303 | 25014.63 |  |  | 16.563 | 4 |
|  | *Dodonaea viscosa* subsp. *angustifolia* | 12754.060 | 1.275 | 7415.15 |  |  | 4.910 | 5 |
| Micha | *Vachellia sieberiana* | 28418.04 | 2.842 | 25373.25 | 135487.61 | 13.55 | 20.97 | 1 |
|  | *Juniperus procera* | 22534.79 | 2.253 | 20120.35 |  |  | 16.63 | 2 |
|  | *Olea europaea* L. subsp. *cuspidata* | 20820.55 | 2.082 | 18589.77 |  |  | 15.37 | 3 |
|  | *Eucalyptus camaldulensis* | 18505.01 | 1.851 | 16522.33 |  |  | 13.66 | 4 |
|  | *Euphorbia abyssinica* | 10482.23 | 1.048 | 9359.13 |  |  | 7.74 | 5 |
| Mekelet | *Vachellia sieberiana* | 72534.16 | 7.25 | 75556.42 | 154505.47 | 15.45 | 46.95 | 1 |
|  | *Olea europaea L.* subsp. *cuspidata* | 35726.86 | 3.57 | 37215.48 |  |  | 23.12 | 2 |
|  | *Dodonaea viscosa* subsp. *angustifolia* | 17908.01 | 1.79 | 18654.18 |  |  | 11.59 | 3 |
|  | *Eucalyptus camaldulensis* | 12138.45 | 1.21 | 12644.22 |  |  | 7.86 | 4 |
|  | *Juniperus procera* | 4363.27 | 0.44 | 4545.07 |  |  | 2.82 | 5 |

d) Top five IVI value-ranked species in each forest patches

| **Patch name** | **Species name** | **RD** | **RF** | **RDO** | **IVI** | **Rank** |
| --- | --- | --- | --- | --- | --- | --- |
| Gerado | *Olea europaea* L. subsp. *cuspidata* | 17.16 | 8.93 | 20.86 | 46.95 | 1 |
|  | *Pittosporum viridiflorum* | 11.95 | 9.68 | 19.08 | 40.71 | 2 |
|  | *Dodonaea viscosa* subsp. *angustifolia* | 22.57 | 10.17 | 4.91 | 37.65 | 3 |
|  | *Vachellia sieberiana* | 6.65 | 10.17 | 18.70 | 35.52 | 4 |
|  | *Juniperus procera* | 4.04 | 2.98 | 16.56 | 23.58 | 5 |
| Micha | *Dodonaea viscosa* subsp. angustifolia | 27.83 | 10.08 | 5.31 | 43.23 | 1 |
|  | *Vachellia sieberiana* | 6.89 | 10.08 | 20.97 | 37.95 | 2 |
|  | *Olea europaea* L. subsp. *cuspidata* | 11.02 | 9.66 | 15.37 | 36.05 | 3 |
|  | *Juniperus procera* | 5.98 | 6.30 | 16.63 | 28.92 | 4 |
|  | *Eucalyptus camaldulensis* | 4.71 | 5.46 | 13.66 | 23.83 | 5 |
| Mekelet | *Vachellia sieberiana* | 21.80 | 15.09 | 46.95 | 83.84 | 1 |
|  | *Dodonaea viscosa* subsp. *angustifolia* | 39.55 | 13.84 | 11.59 | 64.97 | 2 |
|  | *Olea europaea* L. subsp. *cuspidata* | 14.43 | 12.58 | 23.12 | 50.14 | 3 |
|  | *Eucalyptus camaldulensis* | 6.72 | 7.55 | 7.86 | 22.13 | 4 |
|  | *Allophylus abyssinicus* | 2.27 | 8.18 | 0.75 | 11.20 | 5 |

Key: Relative density, RD; Relative dominance, RDO; Relative frequency, RF; Important value index, IVI
