# Supplementary material for: Influence of environmental and anthropogenic factors on forest patch composition and structure in North Wollo Zone, Amhara region, Ethiopia
Source: PLoS One. 2025 Sep 23;20(9):e0332831. doi: 10.1371/journal.pone.0332831 (PMC12456791; doi:10.1371/journal.pone.0332831)
Supplement: S6 File — (DOCX) [file pone.0332831.s006.docx]

**S6 File: Synoptic cover abundance value of species in each community type**

| **Species** | **C1** | **C2** | **C3** | **C4** |
| --- | --- | --- | --- | --- |
| *Acacia decurrens* (J.C.Wendl.) Willd. | 0 | 0.27 | 0.02 | 0.32 |
| *Afrocarpus falcatus* (Thunb.) C.N.Page | 0 | 0.33 | 0.08 | 0 |
| *Albizia gummifera* (J.F.Gmel.) C.A.Sm. | 0 | 0 | 0 | 0.05 |
| *Allophylus abyssinicus* (Hochst.) Radlk. | 0.77 | 0.27 | 1.4 | 0.21 |
| *Arundo donax* L. | 0 | 0 | 0.04 | 0 |
| *Calotropis procera* (Aiton) W.T.Aiton | 0.08 | 0 | 0.02 | 0 |
| *Calpurnia aurea* (Aiton) Benth. | 0.08 | 0 | 0 | 0.05 |
| *Carissa spinarum* L. | 0 | 1.2 | 1 | 0.68 |
| *Casuarina equisetifolia* L. | 0 | 0 | 0 | 0.05 |
| *Celtis africana* Burm.f. | 0.08 | 0 | 0.02 | 0.16 |
| *Citrus aurantiifolia* (Christm.) Swingle | 0 | 0.27 | 0 | 0 |
| *Cordia africana* Lam. | 0 | 0 | 0.1 | 0 |
| *Croton macrostachyus* Hochst. ex Delile | 0 | 0 | 0.04 | 0.16 |
| *Dichrostachys cinerea* (L.) Wight & Arn. | 0.08 | 0 | 0 | 0 |
| *Dodonaea viscosa* subsp. angustifolia (L.f.) J.G.West | 2.92 | 4.93 | 4.21 | 3.47 |
| *Dombeya torrida* (J.F.Gmel.) Bamps | 0.08 | 0.07 | 0.21 | 0.05 |
| *Ehretia cymosa* Thonn | 0 | 0 | 0 | 0.21 |
| *Eucalyptus camaldulensis* Dehnh. | 1.77 | 0.67 | 0.62 | 4.89 |
| *Euclea racemosa* L. | 1.69 | 0.93 | 0.98 | 0.53 |
| *Euphorbia abyssinica* J.F.Gmel. | 0.77 | 0.2 | 1 | 0.42 |
| *Euphorbia tirucalli* L. | 0 | 0 | 0.04 | 0.16 |
| *Faidherbia albida* (Delile) A.Chev. | 0 | 0.07 | 0.08 | 0 |
| *Ficus capreifolia* Delile | 0.31 | 0 | 0 | 0 |
| *Ficus sur* Forssk. | 0 | 0 | 0.02 | 0 |
| *Ficus vasta* Forssk. | 0 | 0 | 0.12 | 0 |
| *Galiniera saxifraga* (Hochst.) Bridson | 0 | 0 | 0.02 | 0.05 |
| *Grevillea robusta* A.Cunn. ex R.Br. | 0 | 0 | 0.08 | 0 |
| *Grewia bicolor* Juss. | 0 | 0 | 0 | 0.05 |
| *Grewia ferruginea* Hochst. ex A.Rich. | 0.23 | 0.13 | 0.12 | 0.63 |
| *Gymnanthemum amygdalinum* (Delile) Sch.Bip. | 0.08 | 0.2 | 0.04 | 0 |
| *Gymnosporia senegalensis* (Lam.) Loes. | 0.08 | 0.4 | 0.54 | 0 |
| *Hesperocyparis lusitanica* (Mill.) Bartel | 1.92 | 0.07 | 0.04 | 0.53 |
| *Heteromorpha arborescens* (Spreng.) Cham. & Schltdl. | 0 | 0.2 | 0.15 | 0 |
| *Jacaranda mimosifolia* D.Don | 0 | 0 | 0 | 0.16 |
| *Juniperus procera* Hochst. ex Endl. | 7 | 0.67 | 1.38 | 0.21 |
| *Moringa oleifera* Lam. | 0 | 0 | 0.02 | 0 |
| *Myrsine africana* L. | 0 | 0.73 | 0.88 | 0.11 |
| *Olea europaea* L. subsp. *cuspidata* (Wall. & G.Don) Cif. | 2.54 | 2.13 | 5.52 | 3.16 |
| *Opuntia ficus-indica* (L.) Mill. | 0 | 0 | 0.06 | 0 |
| *Osyris lanceolata* Hochst. & Steud. | 1 | 0.53 | 0.71 | 0.68 |
| *Pittosporum viridiflorum* Sims | 1.62 | 5.07 | 2.9 | 0.53 |
| *Premna schimperi* Engl. | 0 | 0 | 0.17 | 0 |
| *Pterolobium stellatum* (Forssk.) Brenan | 0 | 0.47 | 0.21 | 0.16 |
| *Rhamnus prinoides* L'Hér. | 0 | 0 | 0.02 | 0 |
| *Rosa abyssinica* R.Br. ex Lindl. | 0 | 0 | 0.04 | 0 |
| *Schinus molle* L. | 0.23 | 0 | 0.08 | 0 |
| *Searsia glutinosa* (Hochst. ex A.Rich.) Moffett | 0.08 | 0.6 | 0.27 | 0 |
| *Searsia retinorrhoea* (Steud. ex Oliv.) Moffett | 0.77 | 3.2 | 0.44 | 0.26 |
| *Senegalia brevispica* (Harms) Seigler & Ebinger | 0 | 0.07 | 0.06 | 0 |
| *Vachellia amythethophylla* (Steud. ex A.Rich.) Kyal. & Boatwr. | 0 | 0 | 0 | 0.32 |
| *Vachellia etbaica* (Schweinf.) Kyal. & Boatwr. | 0.62 | 1.07 | 0.46 | 0.37 |
| *Vachellia seyal* (Delile) P.J.H.Hurter | 0.15 | 0 | 0.15 | 0 |
| *Vachellia sieberiana* (DC.) Kyal. & Boatwr. | 4.08 | 5.4 | 3.15 | 5.63 |
| *Vachellia tortilis* (Forssk.) Galasso & Banfi | 0 | 0 | 0.06 | 0 |
| *Ziziphus spina-christi* (L.) Desf. | 0 | 0.27 | 0.08 | 0.26 |

C1 = Community type 1, C2= Community type 2, C3= Community type 3, C4= Community type 4
