# Supplementary material for: Influence of environmental and anthropogenic factors on forest patch composition and structure in North Wollo Zone, Amhara region, Ethiopia
Source: PLoS One. 2025 Sep 23;20(9):e0332831. doi: 10.1371/journal.pone.0332831 (PMC12456791; doi:10.1371/journal.pone.0332831)
Supplement: S7 File — (DOCX) [file pone.0332831.s007.docx]

**S7 File: Community type classification**

| **Community class** | **Description** | **Details** |
| --- | --- | --- |
| Community type 1  **(C1)** | Total plots and plot numbers | **13** (1, 3,25,2,5,8,26,68,46,49,58,70,71) |
|  | Altitude range | 1925 - 2204 |
|  | Characteristics species | ***Hesperocyparis lusitanica - Juniperus procera*** |
|  | Dominant species | *Juniperus procera, Vachellia sieberiana, Dodonaea viscosa* subsp. *angustifolia, Olea europaea* L. subsp. *cuspidata*, *Hesperocyparis lusitanica, Eucalyptus camaldulensis, Euclea racemosa, Pittosporum viridiflorum* and *Osyris lanceolata.* |
| Community type 2  **(C2)** | Total plots and plot numbers | **19** (14,52,77,93,86,90,87,73,78,94,16,39,45,53,65,72,85,83,84) |
|  | Altitude range | 1929 - 2178 |
|  | Characteristics species | ***Pittosporum viridiflorum - Dodonaea viscosa subsp. angustifolia*** |
|  | Dominant species | *Vachellia sieberiana, Pittosporum viridiflorum****,*** *Dodonaea viscosa* subsp. *angustifolia, Searsia retinorrhoea, Olea europaea* L. subsp. *cuspidata*, *Carissa spinarum, Vachellia etbaica* and *Euclea racemosa.* |
| Community type 3  **(C3)** | Total plots and plot numbers | **12** (4, 6,57,34,51,61,48,44,36,60,63,47) |
|  | Altitude range | 1946 - 2226 |
|  | Characteristics species | ***Olea europaea L. subsp. cuspidate - Allophylus abyssinicus*** |
|  | Dominant species | *Olea europaea* L. *subsp. cuspidata, Dodonaea viscosa* subsp*. angustifolia, Vachellia sieberiana, Pittosporum viridiflorum, Allophylus abyssinicus,* *Juniperus procera, Carissa spinarum, Euphorbia abyssinica* and *Euclea racemosa.* |
| Community type 4  **(C4)** | Total plots and plot numbers | **51**(7,12,37,89,91,11,76,95,15,74,75,79,64,80,69,81,88,9, 24,19,40,55,67,27,82,92,18,20,29,35,42,54,50,59,41,56,31,43,28,32,62,30,38,10,13,22,17,23,21,66,33) |
|  | Altitude range | 1937 – 2413 |
|  | Characteristics species | ***Vachellia sieberiana - Eucalyptus camaldulensis*** |
|  | Dominant species | *Vachellia sieberiana, Eucalyptus camaldulensis, Dodonaea viscosa* subsp. *angustifolia, Olea europaea* L. subsp. *cuspidata, Carissa spinarum, and Osyris lanceolata.* |

**Note:** Plots 1–28 were recorded from the Gerado forest patch, plots 29–71 were recorded from the Gedo forest patch, and plots 72–95 were collected from the Mekelet forest patch.
