# Supplementary figures and images for: Influence of environmental and anthropogenic factors on forest patch composition and structure in North Wollo Zone, Amhara region, Ethiopia
Source: PLoS One. 2025 Sep 23;20(9):e0332831. doi: 10.1371/journal.pone.0332831 (PMC12456791; doi:10.1371/journal.pone.0332831)

**S8 File: Optimal number of clusters in the studied forest patches**


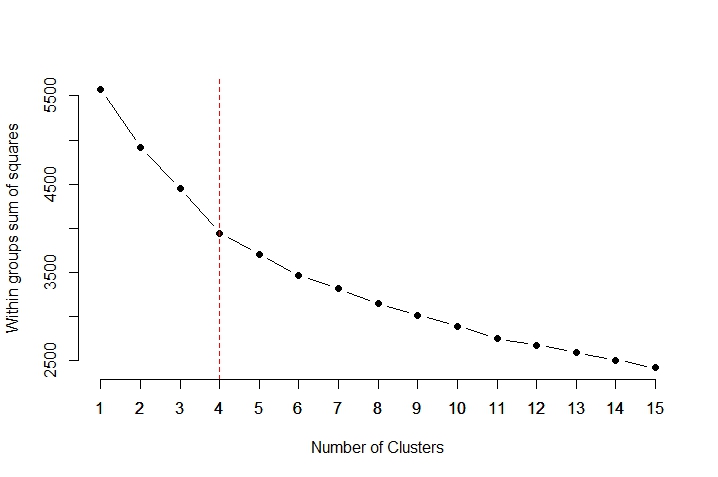

Supplement: S8 File — (DOCX) [file pone.0332831.s008.docx]
