# Supplementary material for: Influence of environmental and anthropogenic factors on forest patch composition and structure in North Wollo Zone, Amhara region, Ethiopia
Source: PLoS One. 2025 Sep 23;20(9):e0332831. doi: 10.1371/journal.pone.0332831 (PMC12456791; doi:10.1371/journal.pone.0332831)

**S9 File: R Code and results for rarefaction and extrapolation analyses**

OPEN=cat("\n PROVIDE FILE NAME OF VEGETATION DATA IN THE APPROPRIATE FOLDER \n")

vegedat<-read.csv(file.choose(), header=TRUE, row.names=1)

str(vegedat)

##Load packages

#library(vegan)

#library(iNEXT)

#library(ggplot2)

#library(devtools)

iNEXT Package

## install iNEXT package from CRAN

install.packages("iNEXT")

## install iNEXT from github

install.packages('devtools')

library(devtools)

install_github('AnneChao/iNEXT')

## import packages

library(iNEXT)

library(ggplot2)

data(spider)

out <- iNEXT(spider, q=c(0, 1, 2), datatype="abundance", endpoint=500)

# Sample-size-based R/E curves, separating plots by "Assemblage"

ggiNEXT(out, type=1, facet.var="Assemblage")

# Sample-size-based R/E curves, separating plots by "Order.q"

ggiNEXT(out, type=1, facet.var="Order.q")

ggiNEXT(out, type=2)

ggiNEXT(out, type=3, facet.var="Assemblage")

ggiNEXT(out, type=3, facet.var="Order.q")

# Separating plots by "order", and display black-white plots

ggiNEXT(out, type=1, facet.var="Order.q", grey=TRUE)

**Output**


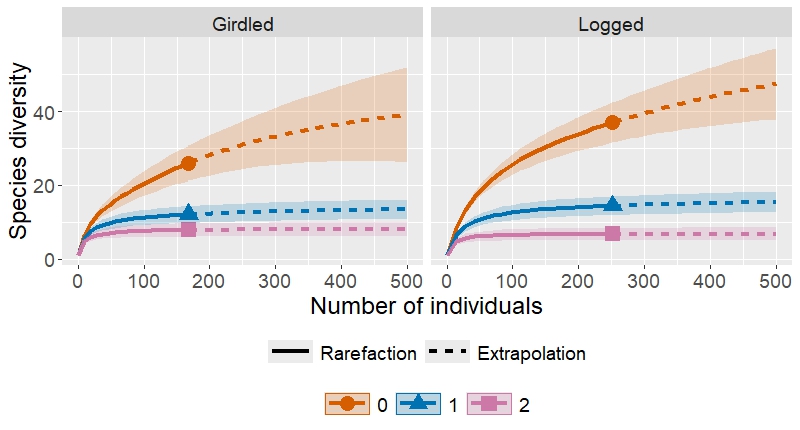

Supplement: S9 File — (DOCX) [file pone.0332831.s009.docx]
